# Applying Na/Co(ii) bimetallic partnerships to promote multiple Co–H exchanges in polyfluoroarenes†

**DOI:** 10.1039/d3cc01216f

**Published:** 2023-04-11

**Authors:** Alessandra Logallo, Eva Hevia

**Affiliations:** a Departement für Chemie, Biochemie und Pharmazie, Universität Bern Bern Switzerland eva.hevia@unibe.ch

## Abstract

Heterobimetallic base NaCo(HMDS)_3_ [HMDS = N(SiMe_3_)_2_] enables regioselective di-cobaltation of activated polyfluoroarenes under mild reaction conditions. For 1,3,5-C_6_H_2_X_3_ (X= Cl, F), NaCo(HMDS)_3_ in excess at 80 °C impressively induces the collective cleavage of five bonds (two C–H and three C–X) of the substrates *via* a cascade activation process that cannot be replicated by LiCo(HMDS)_3_ or KCo(HMDS)_3_.

## Abstract 2

Using a bimetallic base combining Na and Co(ii) enables the regioselective sodium-mediated di-cobaltation of fluoroarenes as well as transforming the three C–F bonds of trifluorobenzene into C–N bonds *via* a cascade activation process.

## Graphical abstract

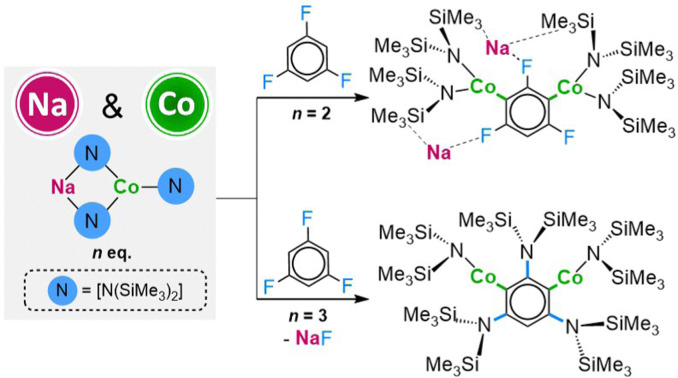

Deprotonative metalation where an inert C–H bond is converted into a more polar, and therefore more reactive, C–metal bond is a fundamentally important transformation in synthesis as a prerequisite for onward functionalisation of aromatic molecules.^1^ While organolithium reagents have traditionally been the reagents of choice to perform these reactions, often they lack good selectivity and exhibit limited functional tolerance even when working under cryogenic conditions so research towards development of improved bases remains a high priority. Main group heterobimetallic systems incorporating both an alkali-metal and a less electropositive metal such as Mg, Zn or Al have emerged as excellent alternatives.^2^ Promoting low polarity metalations, specifically alkali metal mediation (AMM), these systems are compatible with a large variety of functional groups and importantly provide greater stability to the metalated aryl fragments.^3^ Furthermore, in some cases by controlling the stoichiometry of the reaction selective dimetalations can be obtained, as demonstrated by Mulvey for the special *ortho–meta*’ dimagnesiation of substrates such as anisole or trifluorotoluene.^4^ Recent studies have shown that synergic reactivities can also be switched on when pairing an alkali-metal with a divalent earth abundant transition metal (M = Mn, Fe, Co) allowing direct metalation of arenes *via* M–H or M-halogen exchange.^5^ Interestingly, contrasting with other established C–H bond activation processes,^6^ these reactions operate without a change in the oxidation state of the 3d-transition metal.^7^

Within fluoroarene studies sodium cobaltate NaCo(HMDS)_3_ [HMDS = N(SiMe_3_)_2_] (1), in combination with homometallic NaHMDS, has shown particular promise in the regioselective cobaltation of a range of fluoroarenes at room temperature including pentafluorobenzene with excellent atom efficiency to give disodium tetra(aryl) Co(ii) as the THF complex [(THF)_4_Na_2_Co(C_6_F_5_)_4_] (I). In this molecule Co exhibits an unusual square planar geometry (Scheme 1). Mechanistic studies revealed that the reaction occurs with initial formation of [NaCo(HMDS)_2_(C_6_F_5_)] (II) which undergoes ligand redistribution to form I (Scheme 1).^7^ This work concluded that while formally these reactions are cobaltations, sodium plays a key role not only on mediating the metalation but also on the stabilisation of I*via* formation of Na–F interactions. The mild reaction conditions employed and the thermal stability of I contrasts with previous reports on the metalation of fluoroarenes using organolithium reagents which inevitably require use of cryogenic temperatures in order to avoid unwanted side reactions (autometalation, benzyne formation, multimetalation, *etc.*).^8^

**Scheme 1:**
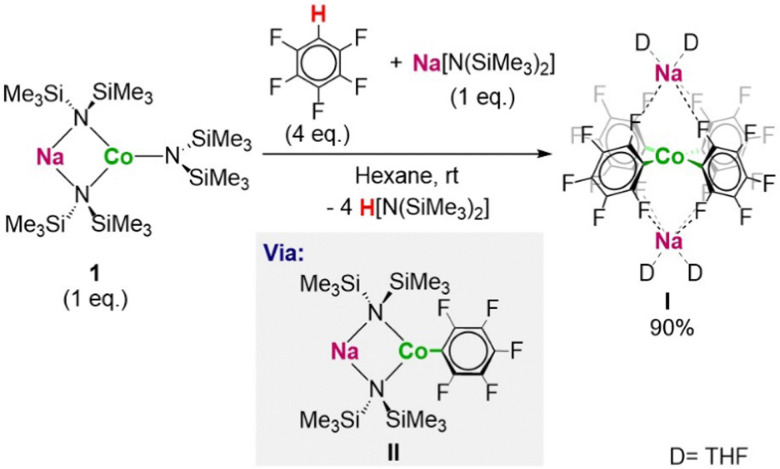
Previous work on metalation of fluoroarenes using the heterobimetallic base NaCo(HMDS)_3_ [HMDS = N(SiMe_3_)_2_] (1).

Recently our group has also reported that by using a two molar excess of a potassium zincate supported by a large sterically demanding silylbis(amide) it is possible to induce dizincation of 1,2,3,4-tetrafluorobenzene. In this case the bulky ligand was proposed to be key, providing steric protection for the trapping of the C_6_F_4_ dianion, although forcing reaction conditions were required (16 h, 70 °C).^9^ Sodium ferrate [(dioxane)NaFe(HMDS)_3_] can also promote the di-ferration of activated fluoroarenes, although the scope of this reactivity has not been fully investigated.^5*a*^ Opening new ground in this area, building on the mechanistic understanding acquired in sodium-mediated cobaltations here we evaluate the potential of 1 to promote dimetalations of fluoro- and chloro-arenes.

We commenced our studies by investigating the reactivity of heterobimetallic NaCo(HMDS)_3_ (1) towards the di-cobaltation of 1,4-difluorobenzene. With a p*K*_a_ of 40.1^10^ its aromatic hydrogens are poorly activated towards metalation. The reaction was carried out in the presence of a 2 molar excess of NaCo(HMDS)_3_ and refluxing conditions (80 °C) for 1 h. Disappointingly, despite the excess of base and forcing conditions, [NaCo(C_6_F_2_H_3_)(HMDS)_2_] (2) was obtained as the only metallated product in a 56% isolated yield. ^1^H NMR monitoring of the reaction showed a conversion of 77% (Fig. 1a). The bimetallic constitution of 2 was established by X-ray crystallographic studies that also confirm the selective *ortho*-cobaltation of the substrate (see ESI†). Remarkably, activation of the remaining HMDS groups to form a tetra(aryl) cobalbate as I was not observed even in the presence of an excess of NaHMDS, which we attribute to the lack of F atoms in the appropriate disposition to stabilise the Na atoms *via* Na⋯F interactions as was a core feature of I.

**Fig. 1:**
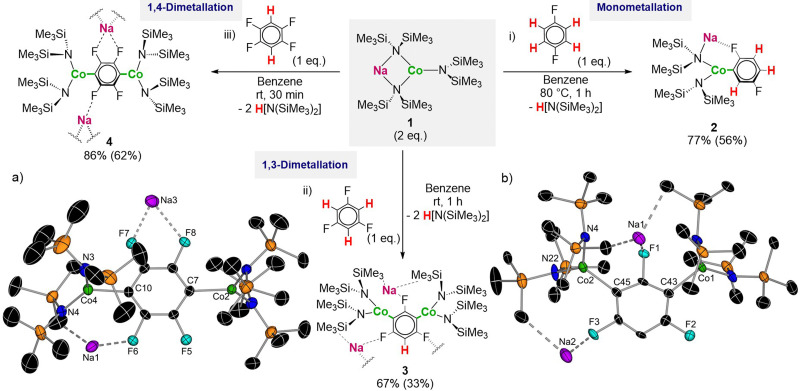
(i) Monometalation of 1,4-difluorobenzene with 2 eq. of 1 to form product 2; (ii) reaction of 1,3,5-trifluorobenzene with 2 eq. of 1 to form the 1,4-dimetalation product 3; (iii) reaction of 1,2,4,5-tetrafluorobenzene with 2 eq. of 1 to form the 1,4-Dimetalation product 4. Conversions determined by ^1^H NMR using hexamethylbenzene as internal standard. Isolated yields shown in brackets. Molecular structures of 3 (b) and 4 (a) with H atoms omitted for clarity and ellipsoids set at 50% probability.

We next decided to try more activated 1,3,5-trifluorobenzene (p*K*_a_ = 31.5).^10^ Remarkably, at room temperature after 1 h selective 1,3 dicobaltation can be observed furnishing [Na_2_Co_2_(C_6_F_3_H)(HMDS)_4_] (3) in a 33% isolated yield (Fig. 1b). Similarly the reaction with 1,2,4,5-tetrafluorobenzene (p*K*_a_ = 23.1)^10^ under the same reaction conditions yielded [Na_2_Co_2_(C_6_F_4_)(HMDS)_4_] (4) in a 62% isolated yield after just 5 minutes at room temperature (Fig. 1c). NMR reaction monitoring in C_6_D_6_ showed that formation of 3 and 4 is quantitative (see ESI†). Note that both dicobaltated products are stable at room temperature for a long time in C_6_D_6_ solutions without observing any degradation. Demonstrating the importance on the choice of base to promote these metalations, while 3 and 4 are formed when using 2 molar excess of 1, if 1,3,5-trifluorobenzene or 1,2,4,5-tetrafluorobenzene are treated with one equivalent of 1 in combination with another equivalent of NaHMDS, which potentially could also promote dimetalation, selective monocobaltation is preferred yielding [Na_2_CoAr_4_] species like I (Scheme 1).^7^ Furthermore, treating these tetra(aryl) cobaltates with another four equivalents of 1 failed to promote a second Co–H exchange even under forcing conditions.

The solid-state structures of 3 and 4 are reminiscent to that of 2, with the Co(ii) centres occupying the positions previously filled by a H atom (Co–C average distance of 2.050 Å for both 3 and 4). In both compounds Na atoms are stabilised by formation of dative bonds with the F substituents. For 3 each Na interacts with a F atom *ortho* to the metalated C (2.244(3) and 2.302(3) Å). In addition, one Na also interacts with the F of a neighbouring unit giving rise to a complex 3D structure (see Fig. S25, ESI†). For 4, a discrete pentameric motif is observed with two Na atoms coordinating to a molecule of benzene (see Fig. S26, ESI†). Both compounds contain medium-long electrostatic interactions between the Na atoms and one or two methyl units belonging to SiMe_3_ groups (ranging from 2.835(5) to of 2.959(7) Å). As far as we can ascertain 3 and 4 constitute the first examples of arene di-cobaltation to be structurally defined. Examples of di-metalated fluoroarenes containing other transition metals are scarce and they are typically obtained *via* C–H or C–F bond activation processes.^11^

To further understanding of these two-fold cobaltations we next assessed the sequential metalation of 1,2,4,5-tetrafluorobenzene (Scheme 2). ^1^H NMR monitoring of the reaction of equimolar amounts of 1 with this fluoroarene in d_8_-Tol solution showed evidence of metalation at 0 °C with the immediate formation of the mono-arene [NaCo(HMDS)_2_(C_6_F_4_H)] (5), which exhibits two distinct resonances at −15.59 ppm and 42.47 ppm (for the HMDS groups and *H*_ortho_ respectively). If the reaction mixture is allowed to reach room temperature, 5 undergoes ligand redistribution to afford the tetra-arene [Na_2_CoAr_4_] (6) (Ar = C_6_F_4_H) with the same structure as I (Scheme 2).^7^ However, when low temperature was maintained and a second equivalent of base 1 was added, an immediate colour change from light turquoise to dark green was observed together with the disappearance of the *H*_ortho_ resonance of 5 and concomitant appearance of a new broad signal at −4.17ppm characteristic of the HMDS groups of the dimetalated product 4 (Scheme 2 and Fig. S26, ESI†). These initial findings prove the ability of heterobimetallic 1 to react with exceptional stoichiometric control *via* a consecutive dimetalation process, where both the mono- and twofold metalated product can be obtained quantitatively under mild reaction conditions.

**Scheme 2:**
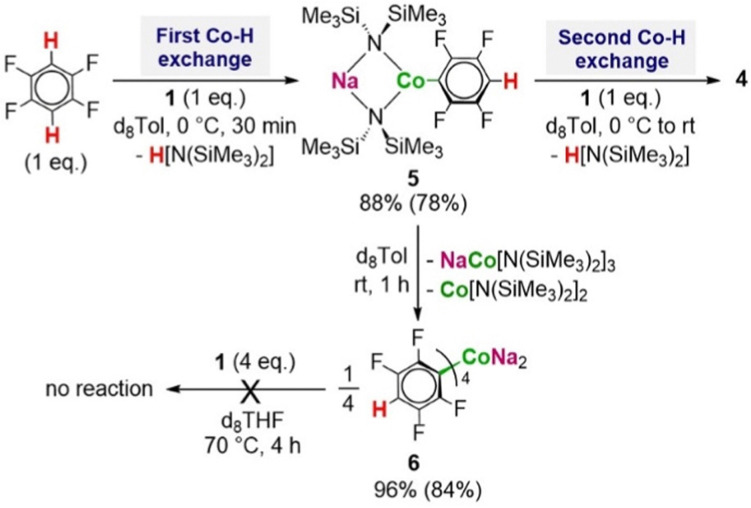
Stepwise di-metalation of 1,2,4,5-tetrafluorobenzene with 2 eq. of (1) to form product (4). Conversion calculated by ^1^H NMR with use of HMB as internal standard. Isolated yields given in brackets.

Building on these findings, we next pondered whether all three aromatic protons of 1,3,5-trifluorobenzene could be activated towards Co–H exchange. Seminal work by Schlosser assessing lithiation of this substrate with a three-molar excess of *t*BuLi at −115 °C in the presence of Me_3_SiCl concluded that only dilithiation species can be formed. In fact, attempts to access a trilithiated product by increasing the temperature to −75 °C led to the formation of 1,3,5-tri-*tert*-butylbenzene.^8*c*^ In our case a 3 : 1 stoichiometry of 1 : 1,3,5-trifluorobenzene was used and the solution was stirred at 80 °C for 16 hours. Re-crystallization from a hexane/THF solution led to the isolation of [1,3-bis(CoHMDS)-2,4,6-tris(HMDS)-C_6_H] (7) in a 78% isolated yield (Fig. 2). X-ray crystallographic studies established the molecular structure of 7 that proved the fluoroarene has undergone a rare two-fold Co–H exchange, 3-fold C–F bond activation event, where three strong C–F bonds^6*b*^ have been replaced by HMDS groups. This reactivity resembles that reported previously by our group for the reaction of the same substrate with the ferrate NaFe(HMDS)_3_ under similar harsh reaction conditions.^5*a*^ Nevertheless, it should be noted that unlike other cobalt mediated C–F bond activations where cobalt changes its oxidation state,^12^7 retains two low coordinate Co(ii) centres. This is also consistent with the measured magnetic moment of 7 (5.56 μB) showing the presence of two *S* = 3/2 centres, akin to what was seen for dimetalated compounds 3 and 4 (see ESI† for details). Each Co in 7 binds to one C of the aromatic ring [2.015(7) and 1.976(7) Å for Co1 and Co2 respectively] as well as engaging in an additional further medium-range contact with the N from a neighbouring HMDS group directly attached to the aryl ring [2.281(6) and 2.340(6) Å for Co1 and Co2 respectively] which induces a considerable distortion from linearity of the N–Co–C angles (156.5(3) and 156.6(3)° for Co1 and Co2 respectively).

**Fig. 2:**
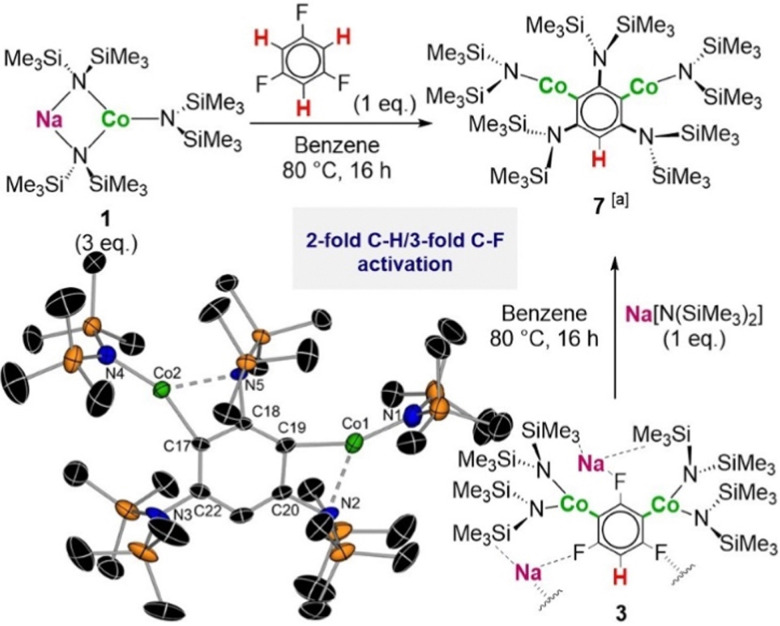
Synthesis of [1,3-(CoHMDS)_2_-2,4,6-(HMDS)_3_-C_6_H] (7). [a] 7 could be obtained from 1,3,5-trifluorobenzene in a 78% isolated yield, 92% isolated yield from 1,3,5-trichlorobenzene and 51% isolated yield from 3 (see ESI†). Molecular structure of 7 with H atoms omitted for clarity and ellipsoids set at 50% probability.

Reactivity studies revealed that while dimetallated complex 3 is stable in solution at room temperature for long periods of time, addition of an extra equivalent of NaHMDS at 80 °C for 16 h affords 7 in quantitative yields. ^1^H NMR monitoring of this reaction showed that along with 7 only a white solid identified as NaF is obtained. Interestingly, no HMDS(H) is detected which suggests the extra equivalent of Na amide is required for the C–F bond activation process but is not involved in a metalation step. Remarkably this reactivity is not available for di-cobaltation product 4 which under the same reaction conditions is completely inert towards the C–F bond activation step. Interestingly 7 could also be obtained when reacting three equivalents of 1 with 1,3,5-trichlorobenzene (93% isolated yield). ^1^H NMR analysis of the reaction crude showed that along with 7 and NaCl, in this case HMDS(H) and Co(HMDS)_2_ are formed. This reactivity marks a significant departure from that of NaFe(HMDS)_3_ which fails to metalate chloroarenes. Based on these findings and the previous studies carried out by Schlosser for the polylithiation of 1,3,5-trifluorobenzene we postulate that 7 is formed as the result of a cascade process (Scheme 3), which under forcing reaction conditions initiates a fast sequence of reactions alternating NaF eliminations with the addition of Co(HMDS)_2_. The fact that other intermediates of this process cannot be trapped or even detected spectroscopically supports that each di-cobaltated intermediate is less stable than precursor 3. The third equivalent of 1 or NaHMDS is required to facilitate the activation of the last F centre. The presence of intramolecular Na⋯F contacts in 3 may also contribute to facilitate these cascade process as previously proposed by Mikami for the C–F bond activation of CF_3_H by lithium enolates.^13^ This was further supported by adding three equivalents of 15-crown-5 to 3, which resulted in the abstraction of the Na cation and formation of a solvent-separated ion pair complex, which is completely inert towards the C–F bond activation processes.

**Scheme 3:**
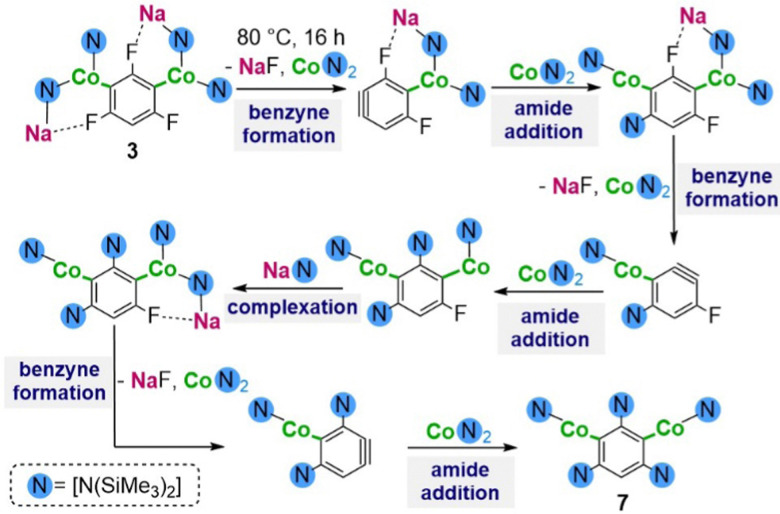
Proposed reaction pathway for the formation of 7.

Finally, an important alkali-metal dependence was noted, with only Na found capable of promoting these transformations. This was discerned on reacting three molar equivalents of MCo(HMDS)_3_ (M = Li, K) with 1,3,5-trifluorobenzene: the Li/Co base only promoted mono-cobaltation of the substrate to form [LiCo(HMDS)_2_(C_6_F_3_H_2_)] (8), and even under forcing reaction conditions (16 h, 80 °C) no evidence of dimetalation was observed; whereas surprisingly the potassium cobaltate is completely inert towards 1,3,5-trifluorobenzene metalation (see ESI† for details). This Li < Na > K trend goes against that normally encountered in group one where reactivity increases in the order Li < Na < K.

To conclude, through isolating key reaction intermediates and NMR reaction monitoring experiments we have revealed the unique ability of sodium cobaltates to promote di-cobaltation of activated fluoroarenes. Under forcing reaction conditions some of these systems can undergo regioselective C–F bond activation *via* a consecutive NaF elimination/Co amide addition cascade process. Assessment of alkali-metal and coordination effects uncovers the key roles of sodium in mediating these Co–H exchanges and transforming C–F into C–N bonds.

We thank the SNSF (projects numbers 206021_177033 and 188573) and the University of Bern for their generous sponsorship of this research.

## Conflicts of interest

There are no conflicts to declare.

## Supplementary Material

CC-059-D3CC01216F-s001

CC-059-D3CC01216F-s002
