# Supplementary material for: Applying Na/Co(ii) bimetallic partnerships to promote multiple Co–H exchanges in polyfluoroarenes
Source: Chem Commun (Camb). 2023 Apr 11;59(36):5383–6. doi: 10.1039/d3cc01216f (PMC10152514; doi:10.1039/d3cc01216f)
Supplement: CC-059-D3CC01216F-s001 [file CC-059-D3CC01216F-s001.pdf]

Electronic Supplementary Material (ESI) for ChemComm.
This journal is © The Royal Society of Chemistry 2023

Supplementary Information for:

Applying Na/Co(II) bimetallic partnerships to promote multiple Co–H exchanges in fluoroarenes

Alessandra Logallo, Eva Hevia*

Department für Chemie und Biochemie, Universität Bern, Freiestrasse 3, 3012 Bern, Switzerland

Contents

Experimental procedures — *3*

General considerations — **3**

Synthesis of numbered compounds — **4**

[NaCo(HMDS)$_2$(C$_6$F$_2$H$_3$)] (2) — 4

[Na$_2$Co$_2$(C$_6$F$_3$H)(HMDS)$_4$] (3) — 4

[Na$_2$Co$_2$(C$_6$F$_4$)(HMDS)$_4$] (4) — 5

[NaCo(HMDS)$_2$(C$_6$F$_4$H)] (5) — 5

[1,3-bis(CoHMDS)-2,4,6-tris(HMDS)-C$_6$H] (7) — 5

[LiCo(HMDS)$_2$(C$_6$F$_3$H$_2$)] (8) — 6

NMR Spectra — **7**

NMR Studies/ Supplementary Experiments — **14**

Stepwise dimetalation of 1,2,4,5-tetrafluorobenzene: — 14

Formation of 7 from 1,3,5-trifluororobenzene: — 15

Formation of 7 from 1,3,5-trichlorobenzene: — 16

Reaction of 3 with 15-crown-5: — 17

NMR Conversions — 18

X-Ray Crystallographic Figures and Data — **29**

X-Ray Tables — **35**

References — *37*

Experimental procedures

General considerations

All reactions were carried out under an inert dry argon atmosphere utilising standard Schlenk line and glove-box techniques (MBraun, MB10 Compact or UniLab Pro, <0.5 ppm H_2O, O_2).

Na(HMDS) was purchased from Sigma Aldrich and used as received.

$Co(HMDS)_2$ and $[NaCo(HMDS)_3]$ (**1**) were prepared according to a modified literature procedure from Lappert *et al.* and Layfield *et al.* respectively.[1-2]

Hexane, toluene, benzene and pentane were dried prior to synthesis by Grubbs column[3] (MBraun SPS 5) to remove any traces of moisture and dissolved oxygen and then further dried by stirring over $NaK_{2.8}$ overnight before vacuum transferring and subsequently storing over 4 Å molecular sieves.

1,4-difluorobenzene, 1,3,5-trifluorobenzene, 1,2,4,5-tetrafluorobenzene and 1,3,5-trichlorobenzene were purchased from either Sigma Aldrich or Fluorochem and were dried by distillation over CaH_2 under a nitrogen or argon atmosphere and stored over 4 Å molecular sieves.

Deuterated solvents C_6D_6, d_8-toluene and d_8-THF for NMR spectroscopy were dried over $NaK_{2.8}$, vacuum transferred and stored over 4 Å molecular sieves in the glove-box prior to use. All NMR spectroscopy samples were prepared inside the inert argon atmosphere of the glove-box.

^{1}H (300.13 MHz), NMR spectra were recorded on a Bruker Avance III HD 300 spectrometer at 300 K and analysed using TopSpin (v4.0.5, Bruker Biospin, Karlsruhe). NMR spectra were referenced internally to the corresponding residual protio solvent peaks.

The paramagnetic nature of the Co(II) compounds here reported precludes the acquisition of ^{19}F and ^{13}C spectra. In particular, no resonances could be observed in the ^{19}F{1H} (376.40 MHz) between 500 and −500 ppm and this is likely due to very rapid relaxation of the ^{19}F nuclei.[4]

Solution magnetic susceptibilities were determined by the Evans method at 300 K.[5] CHN elemental microanalyses were performed on a Flash 2000 Organic Elemental Analyser (Thermo Scientific).

Synthesis of numbered compounds

[NaCo(HMDS)$_2$(C$_6$F$_2$H$_3$)] **(2)**

0.189 g (0.5 mmol) of Co(HMDS)$_2$ and 0.092 g (0.5 mmol) of Na(HMDS) were dissolved in 10 ml of hexane and the bright green solution was left to stir for one hour before removing all solvent under vacuum. The light green solid was redissolved in 5 ml of benzene to which 0.024 ml (0.25 mmol) of 1,4-difluorobenzene were added. The green solution was stirred for 1 h at 80 °C resulting a darkening of the initial colour. Dark green crystals were obtained from a hexane/THF solution, isolated, washed with cold hexane and dried (0.073 g, 56 % yield).

^{1}H NMR (C$_6$D$_6$, 300 K) δ (ppm) = 39.90 [aryl CH] , 9.65 [THF], 3.87 [THF], −2.92 [aryl CH], −18.16 [-SiMe$_3$]

Anal. Calcd for C$_{18}$H$_{39}$CoF$_2$N$_2$NaSi$_4$·1THF: C 44.95, H 8.06, N 4.77 Found: C 45.13, H 8.03, N 4.38

Solution Magnetic Moment (C$_6$D$_6$, 300 K) = 4.36 μ_B

NB: Both ^{1}H NMR and CHN analysis show the retainment of 1·THF.

[Na$_2$Co$_2$(C$_6$F$_3$H)(HMDS)$_4$] (3)

0.189 g (0.5 mmol) of Co(HMDS)$_2$ and 0.092 g (0.5 mmol) of Na(HMDS) were dissolved in 10 ml of hexane and the bright green solution was left to stir for one hour before removing all solvent under vacuum. The light green solid was redissolved in 5 ml of benzene to which 0.026 ml (0.25 mmol) of 1,3,5-trifluorobenzene were added. The green solution was stirred for 1 h at 80 °C. Dark green crystals were obtained from a hexane/THF solution, isolated, washed with cold hexane and dried (0.053 g, 33 % yield).

^{1}H NMR (C$_6$D$_6$, 300 K) δ (ppm) = 317.08 [aryl CH], −15.65 [-SiMe$_3$]

^{1}H NMR (d$_8$-THF, 300 K) δ (ppm) = 296.76 [aryl CH], −13.45 [-SiMe$_3$]

Solution Magnetic Moment (C$_6$D$_6$, 300 K) = 5.69 μ_B

NB: Despite multiple attempts, repeated crystalline samples showed low C combustion values. Anal. Calcd for C$_{18}$H$_{37}$Co$_2$F$_3$N$_4$Na$_2$Si$_4$: C 38.52, H 7.87, N 5.99 Found C 36.67, H 7.15, N 5.42

[$Na_2Co_2(C_6F_4)(HMDS)_4$] (4)

0.189 g (0.5 mmol) of $Co(HMDS)_2$ and 0.092 g (0.5 mmol) of NaHMDS were dissolved in 10 ml of hexane and the bright green solution was left to stir for one hour before removing all solvent under vacuum. The light green solid was redissolved in 5 ml of benzene to which 0.028 ml (0.25 mmol) of 1,2,4,5-tetrafluorobenzene were added forming a dark green solution with a light green precipitate. THF was added dropwise to get an almost clear dark green solution which was heated gently and left to slowly cool down to room temperature. Green crystals were isolated, washed with cold hexane and dried (0.148 g, 62 % yield).

^{1}H NMR (C_6D_6, 300 K) δ (ppm) = −5.02 [$SiMe_3$]

Anal. Calcd for $C_{30}H_{72}Co_2F_4N_4Na_2Si_8$: C 37.79, H 7.61, N 5.88 Found: C 37.81, H 7.27 , N 5.81

Solution Magnetic Moment (C_6D_6, 300 K) = 5.61 μ_B

[$NaCo(HMDS)_2(C_6F_4H)$] (5)

0.189 g (0.5 mmol) of $Co(HMDS)_2$ and 0.092 g (0.5 mmol) of Na(HMDS) were dissolved in 10 ml of toluene and the bright green solution was left to stir for one hour before cooling down to 0 °C. 0.056 ml (0.5 mmol) of 1,2,4,5-tetrafluorobenzene were added. The green solution was stirred for 30 minutes at 0 °C before removing all solvent under vacuum. Cold (-30 °C) hexane was added and the light green solid isolated, washed and dried (0.21 g, 78 % yield).

^{1}H NMR (d_8-Tol, 300 K) δ (ppm) = −15.71 [$SiMe_3$], 42.77 [aryl CH]

Anal. Calcd for $C_{18}H_{37}CoF_4N_2NaSi_4$: C 39.18, H 6.76, N 5.08 Found: C 39.29, H 6.73, N 4.93

Solution Magnetic Moment (d_8-Tol, 300 K) = 4.87 μ_B

[1,3-bis(CoHMDS)-2,4,6-tris(HMDS)-C_6H] (7)

<u>SYNTHESIS FROM 1,3,5-TRIFLUOROBENZENE:</u>

0.284 g (0.75 mmol) of $Co(HMDS)_2$ and 0.137 g (0.75 mmol) of Na(HMDS) were dissolved in 15 ml of hexane and the bright green solution was left to stir for one hour before removing all solvent under vacuum. The light green solid was redissolved in 10 ml of benzene to which 0.026 ml (0.25 mmol) of 1,3,5-trifluorobenzene were added. The dark green solution was refluxed for 16 h at 60 °C before removing all solvent under vacuum. THF was added to a light green hexane suspension to get an almost clear dark green solution which was heated gently and left to slowly cool down to room temperature. Green crystals were isolated, washed with cold hexane and dried (0.074 g, 37 % yield). Alternatively, **7** could be isolated by precipitation in cold (−30 °C) hexane. The light green solid isolated, washed and dried (0.156 g, 78 % yield).

<u>SYNTHESIS FROM 1,3,5-TRICHLOROBENZENE:</u>

0.284 g (0.75 mmol) of $Co(HMDS)_2$ and 0.137 g (0.75 mmol) of Na(HMDS) were dissolved in 15 ml of hexane and the bright green solution was left to stir for one hour before removing all solvent under vacuum. The light green solid was redissolved in 10 ml of benzene to which 0.36 g (0.25 mmol) of

1,3,5-trichlorobenzene were added. The dark green solution was refluxed for 16 h at 60 °C before removing all solvent under vacuum. Cold (-30 °C) hexane was added and the light green solid isolated, washed and dried (0.186 g, 93 % yield).

SYNTHESIS FROM **3**:

0.233 g (0.25 mmol) of **3** and 0.046 g (0.25 mmol) of Na(HMDS) were dissolved in benzene.The dark green solution was refluxed for 16 h at 60 °C before removing all solvent under vacuum. Cold (-30 °C) hexane was added and the light green solid isolated, washed and dried (0.102 g, 51 % yield).

^{1}H NMR (C_6D_6, 300 K) δ (ppm) = 391.39 [aryl CH (C4)], 10.40 [-$SiMe_3$, Co-HMDS or HMDS (4,6)], −16.47[-$SiMe_3$, Co-HMDS or HMDS (4,6)], −80.18 [-$SiMe_3$, HMDS (2)]

Anal. Calcd for $C_{27}H_{64}Co_2N_7Si_7$: C 43.55, H 9.24, N 7.05 Found: C 42.91, H 8.92 , N 6.33

Solution Magnetic Moment (C_6D_6, 300 K) = 5.56 μ_B

[LiCo(HMDS)$_2$($C_6F_3H_2$)] **(8)**

0.284 g (0.75 mmol) of Co(HMDS)$_2$ and 0.125 g (0.5 mmol) of LiHMDS were dissolved in 15ml of hexane and the bright green solution was left to stir for one hour before removing all solvent under vacuum. The light green solid was redissolved in 10 ml of benzene to which 0.025 ml (0.25 mmol) of 1,3,5-trifluorobenzene were added forming an immediate light green solution with light green precipitate. THF was added dropwise to obtain an almost clear solution which was heated and left to slowly cool down to room temperature. Plate-like green crystals were isolated, washed with cold hexane and dried (0.084 g, 65 % yield). Alternatively, **8** could be isolated by precipitation in cold (−30 °C) hexane. The light green solid isolated, washed and dried (0.075 g, 58 % yield).

^{1}H NMR (d_8-THF, 300 K) δ (ppm) = 172.91 [aryl CH] , −16.69 [-$SiMe_3$]

Anal. Calcd for $C_{18}H_{38}CoF_3LiN_2Si_4$: C 41.76, H 7.40, N 5.41 Found C 41.54, H 7.34, N 5.30

Solution Magnetic Moment (C_6D_6, 300 K) = 4.30 μ_B

NMR Spectra

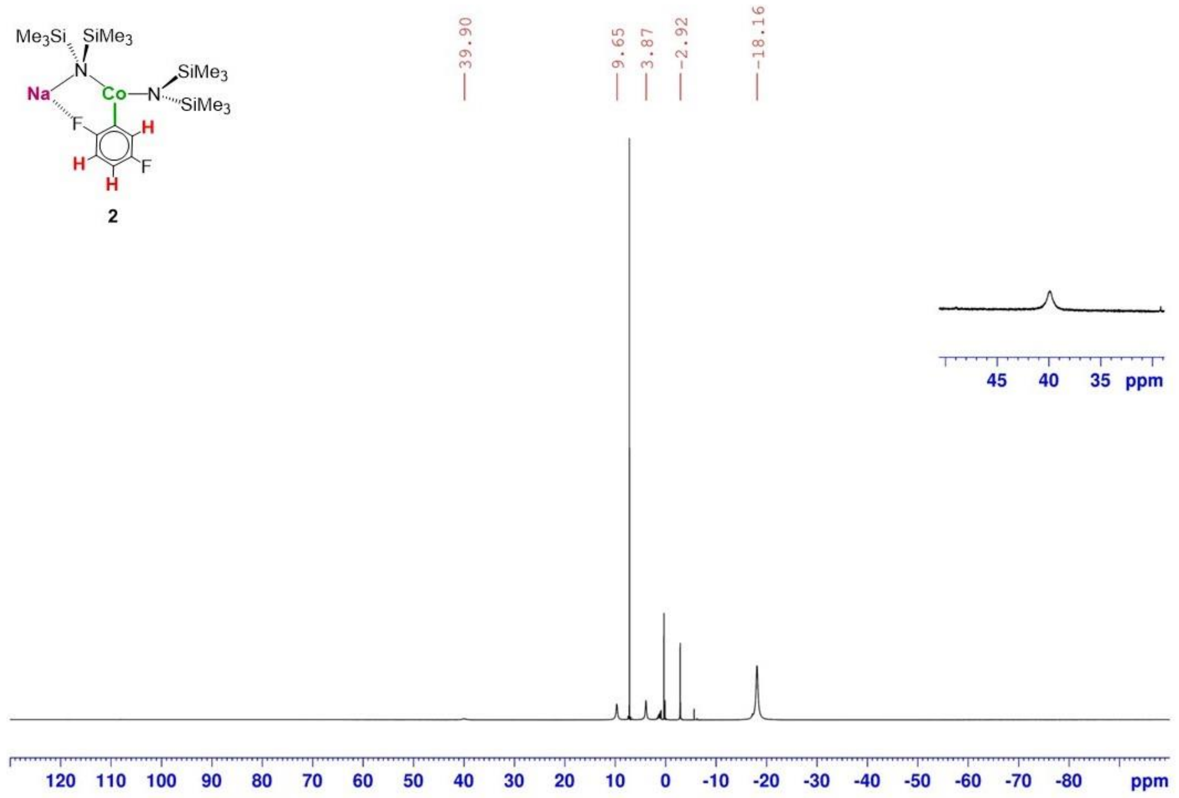

Figure S1. ^{1}H NMR spectrum of **2** in C_6D_6. Note: residual HMDS(H) at 0.76 ppm.

**δ (^{1}H) / ppm**	**Assignment**
39.90	[aryl CH]
9.65	[THF]
3.87	[THF]
−2.92	[aryl CH]
−18.16	[-$SiMe_3$]

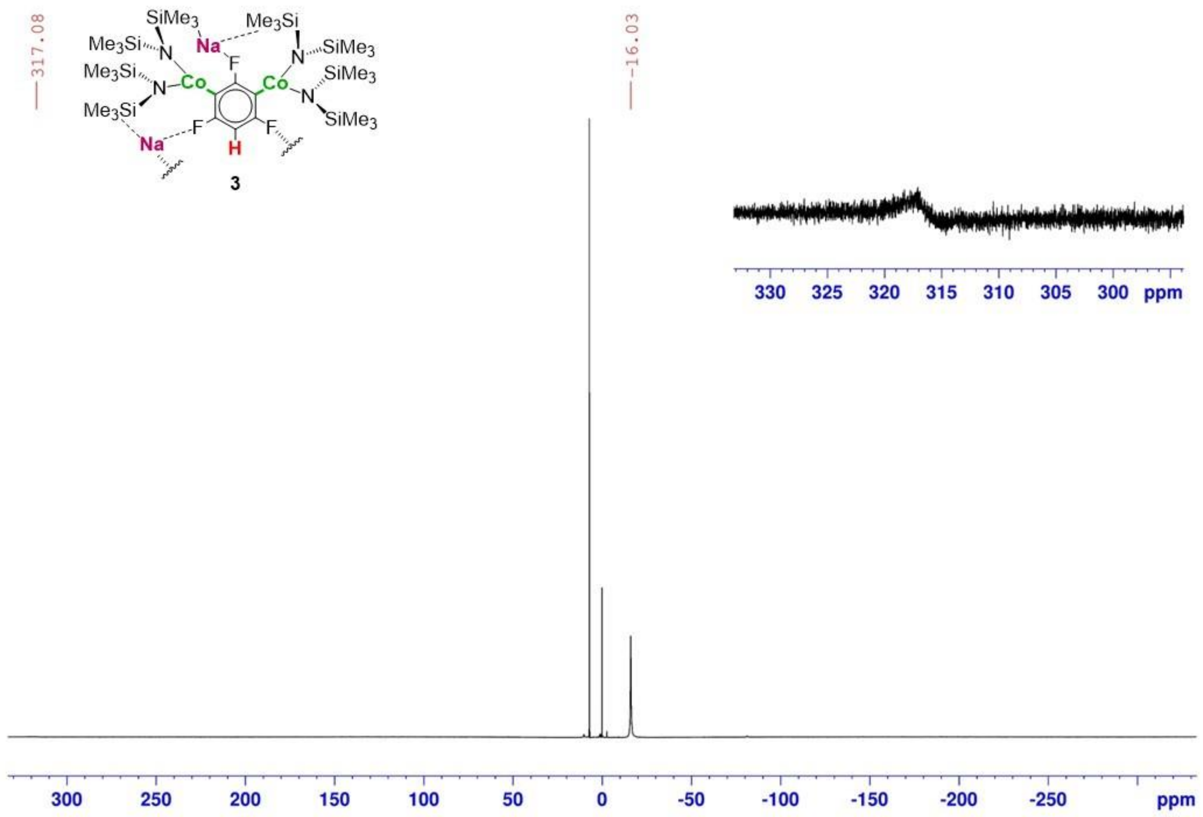

Figure S2. ^{1}H NMR spectrum of **3** in C_6D_6. Note: residual HMDS(H) at 0.76 ppm.

**δ (^{1}H) / ppm**	**Assignment**
317.08	[aryl CH]
−15.65	[-SiMe$_3$]

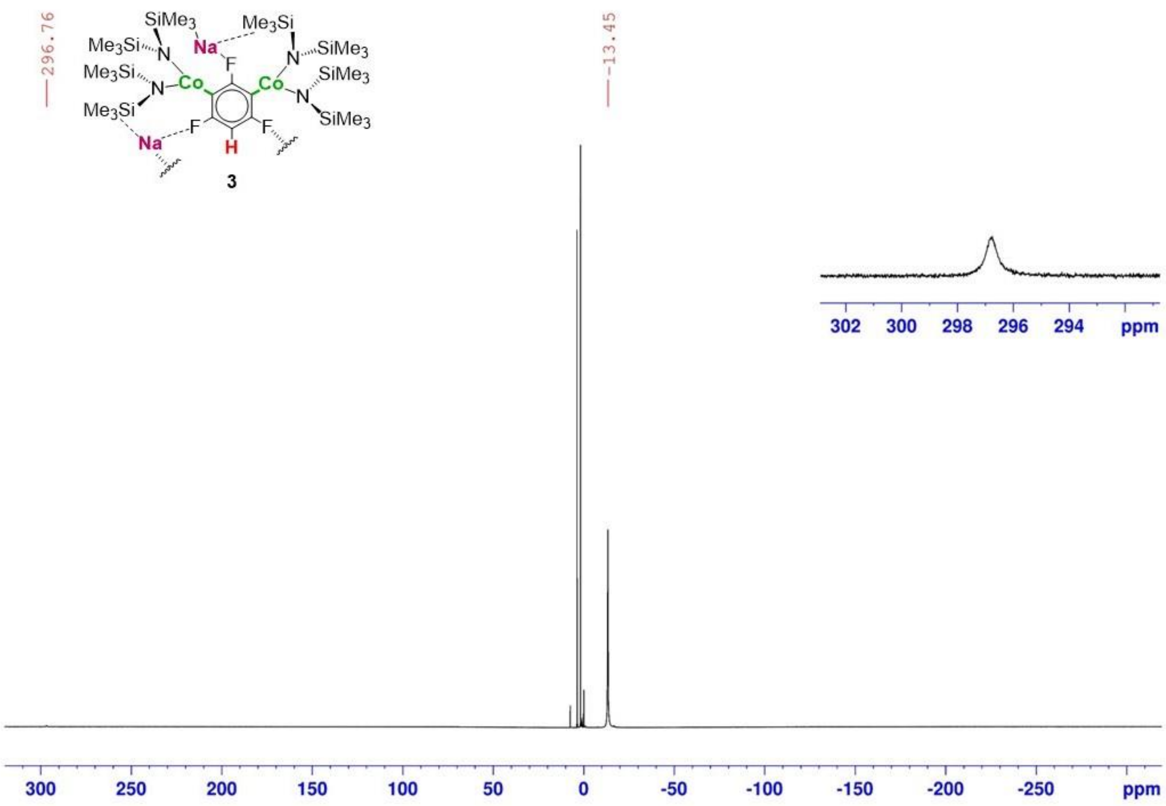

Figure S3. ^{1}H NMR spectrum of **3** in d_8-THF.

**δ (^{1}H) / ppm**	**Assignment**
296.76	[aryl CH]
−13.45	[-$SiMe_3$]

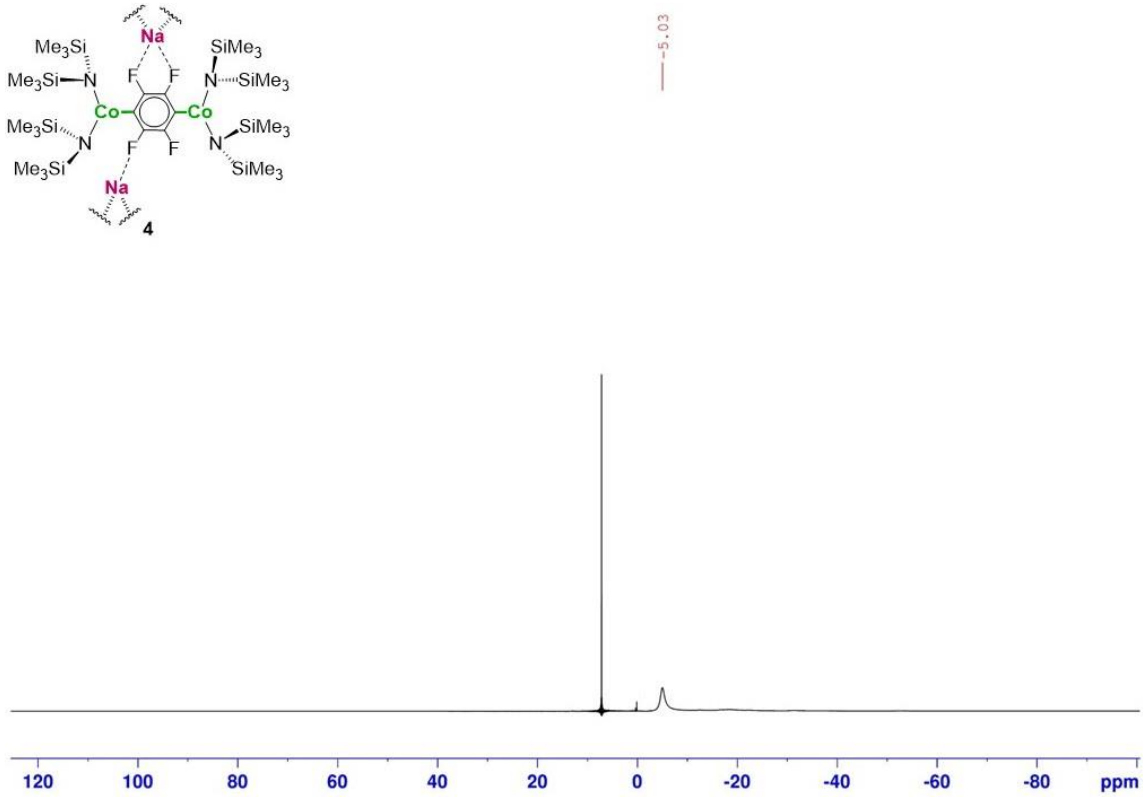

Figure S4. ^{1}H NMR spectrum of **4** in C_6D_6.

**δ (^{1}H) / ppm**	**Assignment**
−5.03	[-$SiMe_3$]

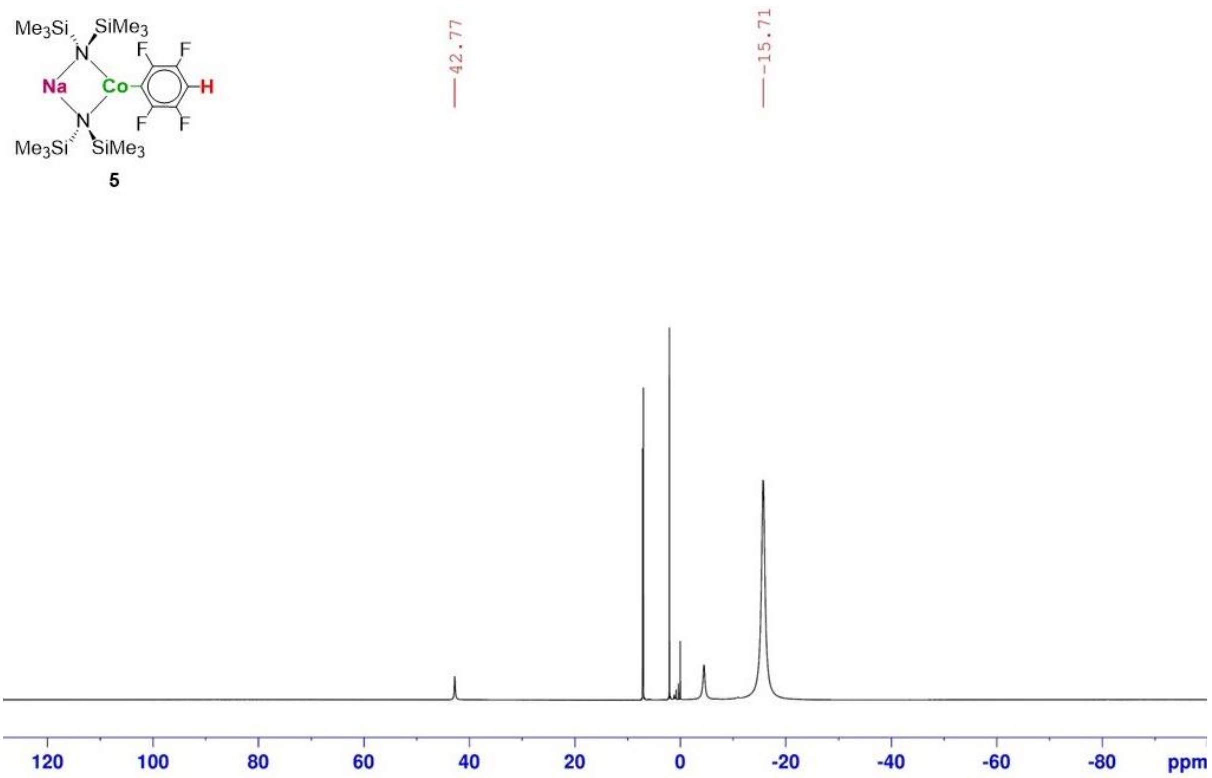

Figure S5. ^{1}H NMR spectrum of **5** in d_8-Tol. Note: residual HMDS(H) at 0.76 ppm.

**δ (^{1}H) / ppm**	**Assignment**
−15.71	[$SiMe_3$]
42.77	[aryl CH]

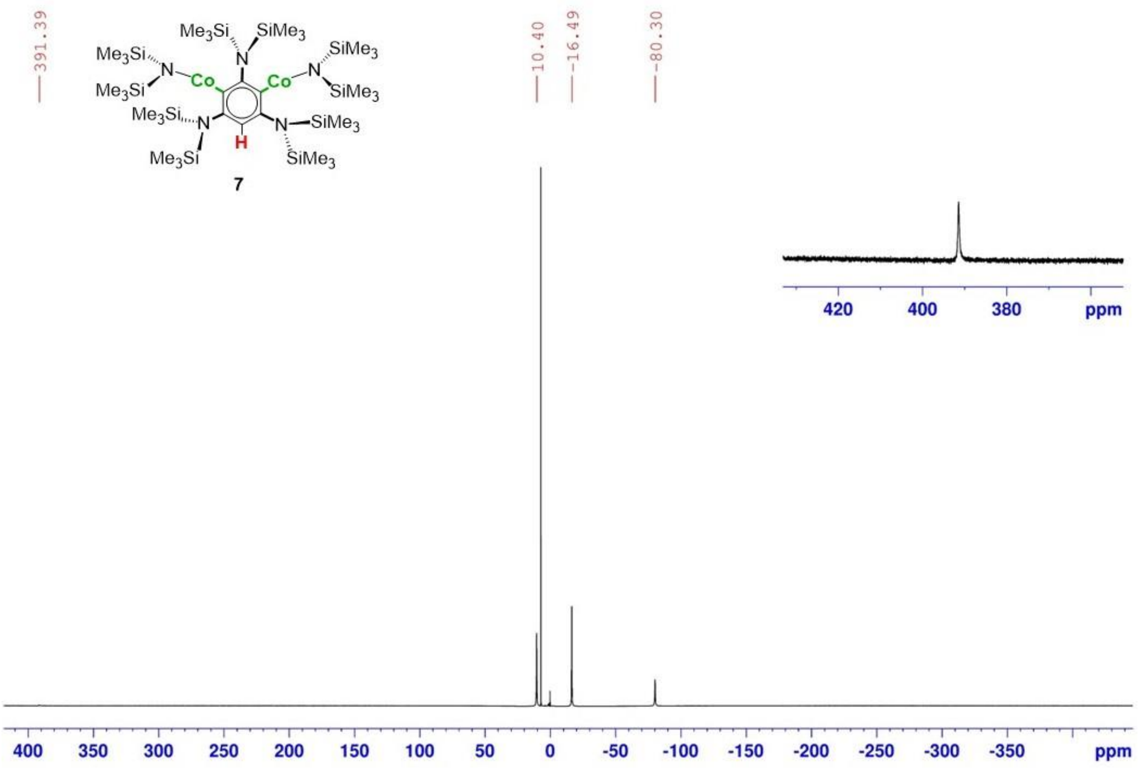

Figure S6. ^{1}H NMR spectrum of **7** in C_6D_6.

**δ (^{1}H) / ppm**	**Assignment**
391.39	[aryl CH (C4)]
10.40	[-$SiMe_3$, Co-HMDS or HMDS (4,6)]
−16.47	[- $SiMe_3$, Co-HMDS or HMDS (4,6)]
−80.18	[-$SiMe_3$, HMDS (2)]

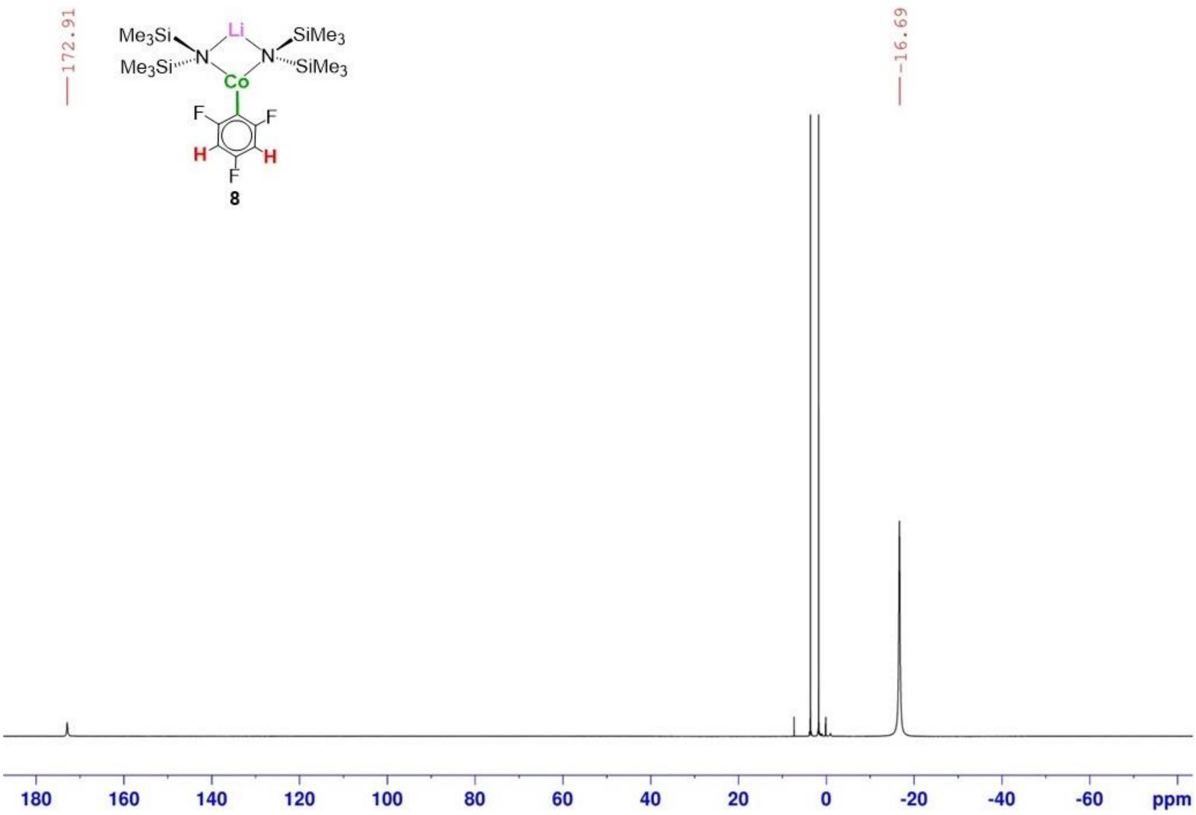

Figure S7. ^{1}H NMR spectrum of **8** in d_8-THF.

δ (^{1}H) / ppm	Assignment
172.91	[aryl CH]
−16.69	[-$SiMe_3$]

NMR Studies/ Supplementary Experiments

Stepwise dimetalation of 1,2,4,5-tetrafluorobenzene:

In a J. Young's NMR tube 1,3,5-tetrafluorobenzene (0.1 mmol, 11 μL) was added to d_8-Tol and the solution cooled to 0 °C. $NaCo(HMDS)_3$ (**1**) (0.1 mmol, 0.056 g) was added, resulting in the formation of a dark green solution. The reaction was monitored by ^1H NMR spectroscopy, which revealed immediate reaction between the two species to form **5** (two broad signals at 42.60 and −15.68 ppm). Whilst still at 0 °C, a second equivalent of **1** (0.1 mmol, 0.056 g) was added. No change in colour was observed, although ^1H NMR showed the disappearance of the peaks of **5** together with the appearance of a new signal at −4.17 which can be attributed to compound **4**.

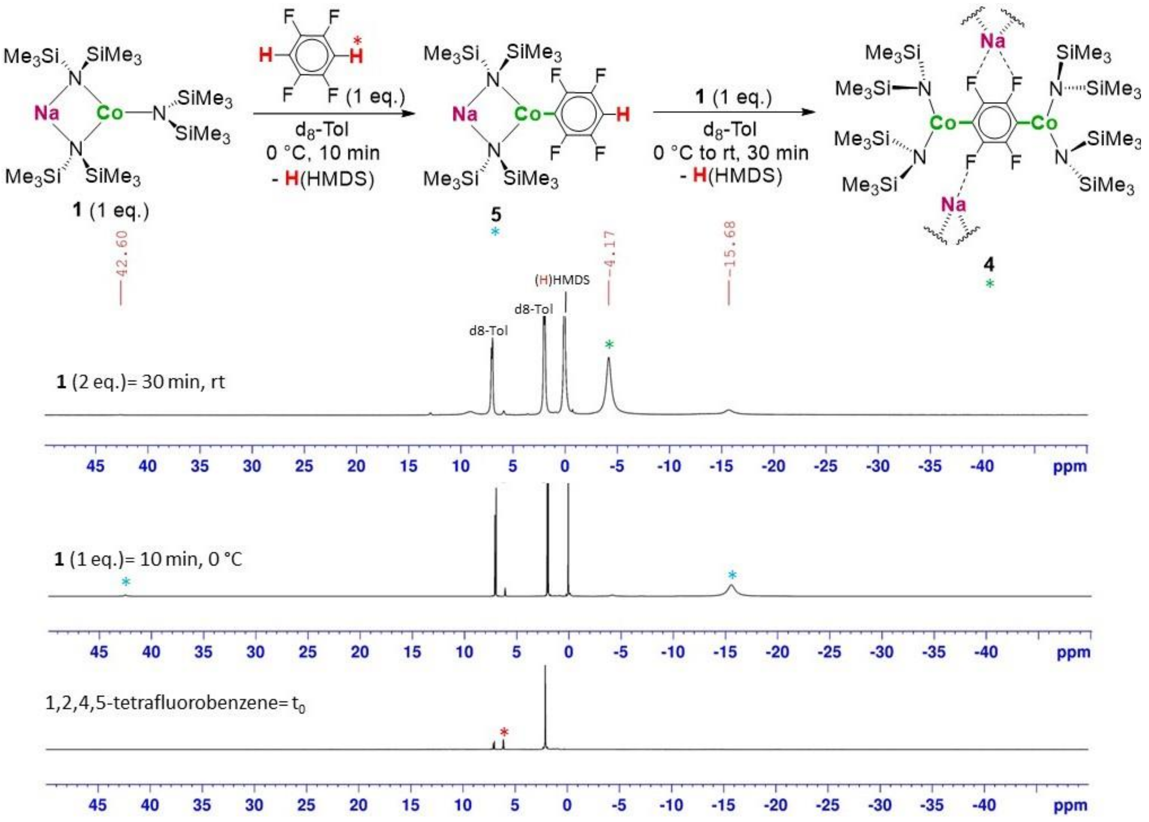

Figure S8. ^1H NMR spectrum of the stepwise dimetalation of 1,2,4,5-tetrafluorobenzene in d_8-Tol.

Formation of **7** from 1,3,5-trifluororobenzene:

In a J. Young's NMR tube 1,3,5-trifluorobenzene (0.1 mmol, 10 μL) was added together with a constant amount of Hexamethylbenzene as internal standard and dissolved in 0.5 mL of C_6D_6. An initial ^{1}H NMR was recorded. To the clear solution 2 eq. of $NaCo(HMDS)_3$ (**1**) (0.2 mmol, 0.112 g) was added to obtain a light green solution. A second ^{1}H NMR was recorded showing a partial consumption of the fluoroarene and the formation of **3**. NaHMDS (0.1 mmol, 0.018 g) was added and the green solution heated at 80 °C for 16 h, causing a visible darkening of the solution from green to light brown. The ^{1}H NMR showed full conversion of 1,3,5-trifluorobenzene and formation of new peaks that can be attributed to compound **7** together with residual peaks of **3** as well as unidentified new paramagnetic species.

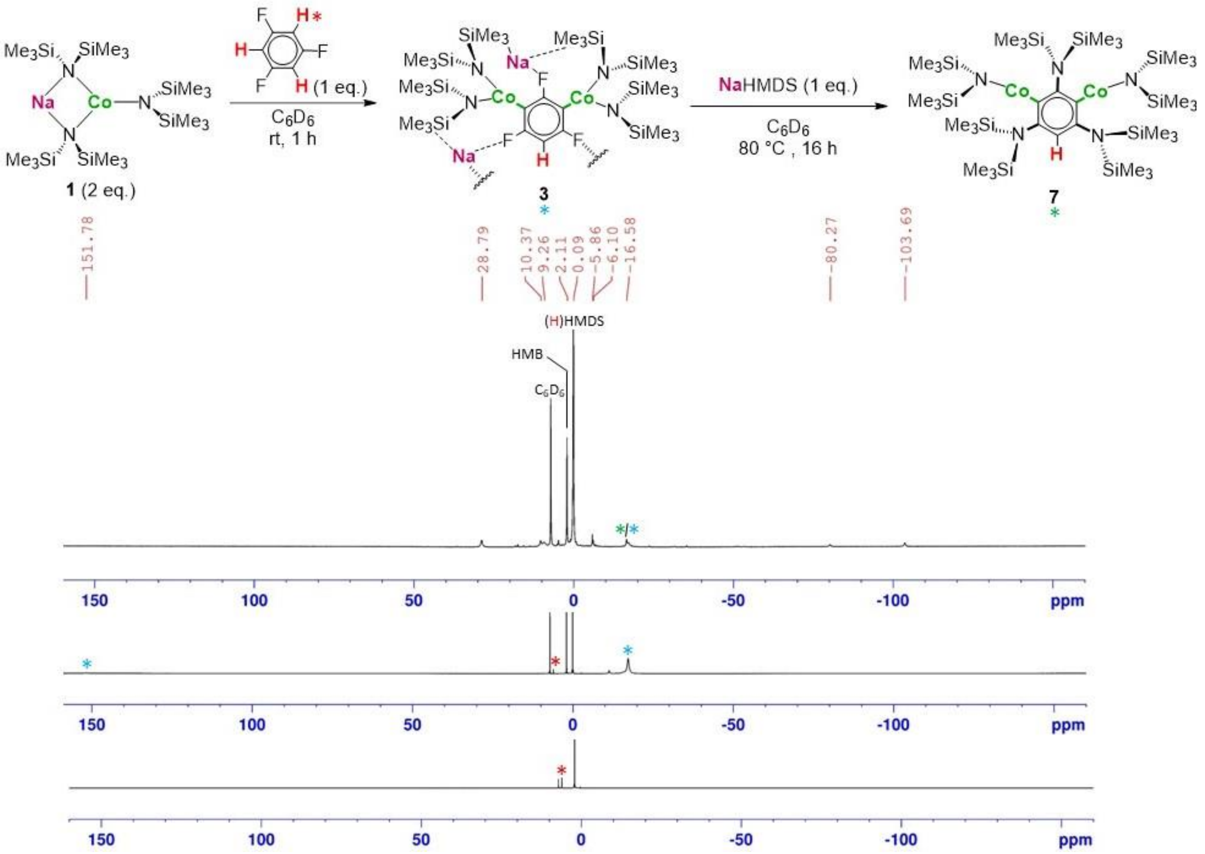

Figure S9. ^{1}H NMR spectrum showing the evolution from 1,3,5-trifluorobenzene to **7** in C_6D_6.

<u>Formation of **7** from 1,3,5-trichlorobenzene:</u>

In a J. Young's NMR tube 1,3,5-trichlorobenzene (0.1 mmol, 0.018 g) was added together with a constant amount of Hexamethylbenzene as internal standard and dissolved in 0.5 mL of C_6D_6. An initial ^1H NMR was recorded. To the clear solution 3 eq. of $NaCo(HMDS)_3$ (**1**) (0.3 mmol, 0.169 g) was added and the green solution heated at 80 °C for 16 h, causing a visible darkening of the solution from green to light brown. The ^1H NMR showed a 51 % conversion of 1,3,5-trichlorobenzene to **7** and other unidentified paramagnetic species. Notably, $Co(HMDS)_2$ (*vbs* at 9.27 ppm) was detected as well as the formation of (H)HMDS.

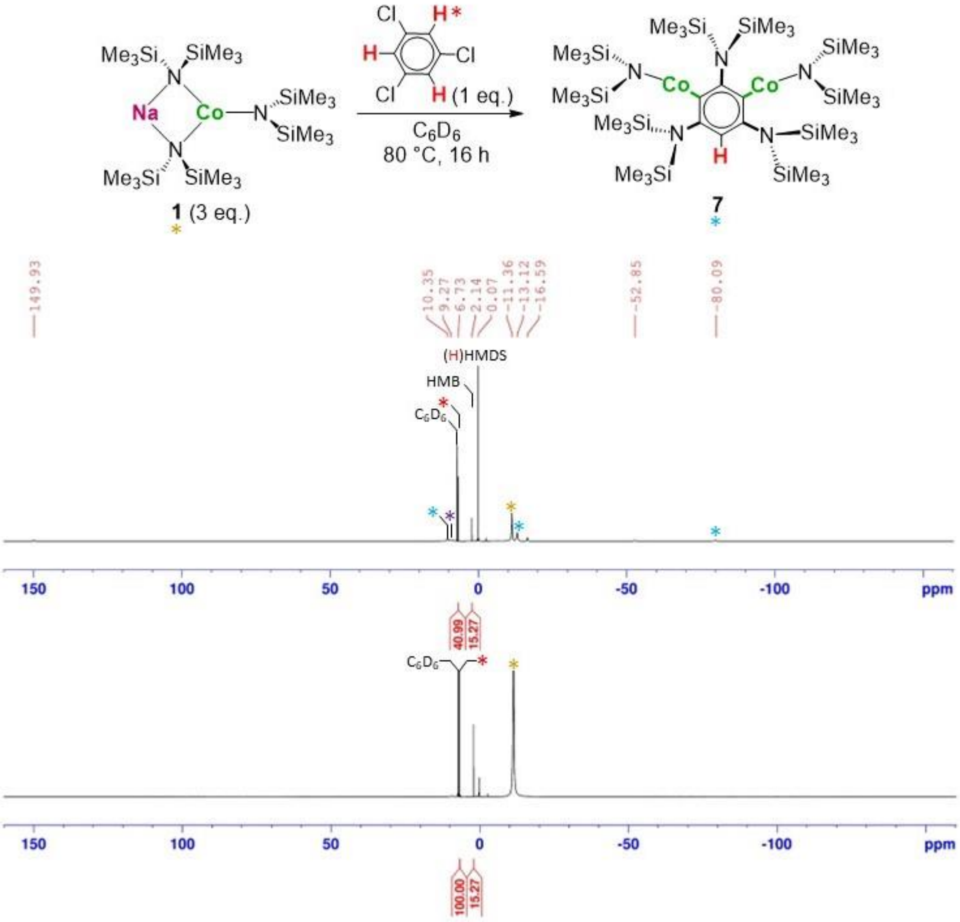

Figure S10. ^1H NMR spectrum showing the Formation of **7** from 1,3,5-trichlorobenze in C_6D_6.

Reaction of **3** with 15-crown-5:

In a J. Young's NMR tube, $[Na_2Co_2(C_6F_3H)(HMDS)_4]$ (**3**) (0.1 mmol, 0.093 g) was dissolved in C_6D_6 and a first ^1H NMR recorded. 15-crown-5 (0.3 mmol, 59 μL) was added to the light green solution causing an immediate colour change to turquoise. The solution was heated at 75 °C for 16 h. A second ^1H NMR was recorded showing the appearance of a new set of paramagnetic signals ranging between 326.59 to −57.12 ppm. Despite all attempts of crystallization of the newly formed paramagnetic species were unsuccessful, it was clear that the formation of compound **7** was completely inhibited.

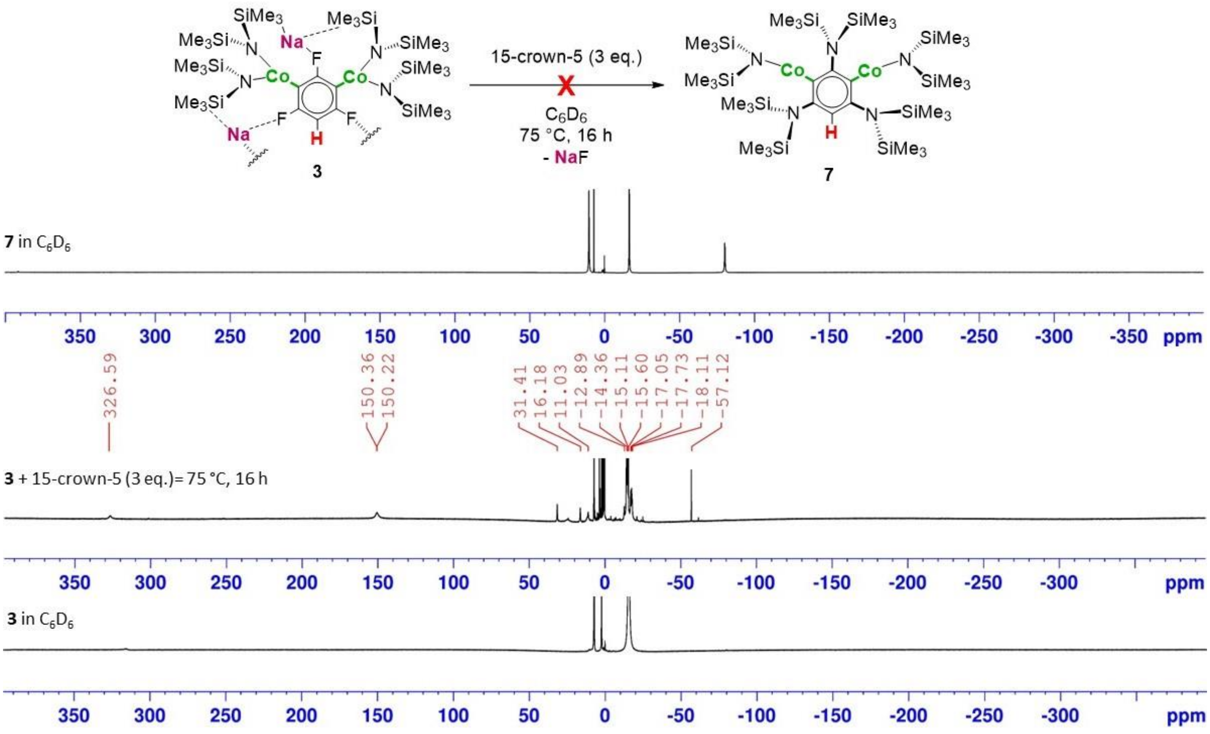

Figure S11. ^1H NMR spectrum of reaction between $[Na_2Co_2(C_6F_3H)(HMDS)_4]$ (**3**) and 15-crown-5.

NMR Conversions

Conversion of 1,4-difluorobenzene to **2**:

In a J. Young's NMR tube 1,4-difluorobenzene (0.1 mmol, 10 μL) was added together with a constant amount of Hexamethylbenzene as internal standard and dissolved in 0.5 mL of C_6D_6. An initial ^1H NMR was recorded. To the clear solution 2 eq. of $NaCo(HMDS)_3$ (**1**) (0.2 mmol, 0.112 g) was added to obtain a light green solution. After heating for 1 hour at 80 °C a second ^1H NMR was recorded showing a final 77 % conversion of 1,4-difluorobenzene to $[NaCo(HMDS)_2(C_6F_2H_3)]$ (**2**). The second equivalent of **1** can be observed unreacted in solution (−11.06 ppm).

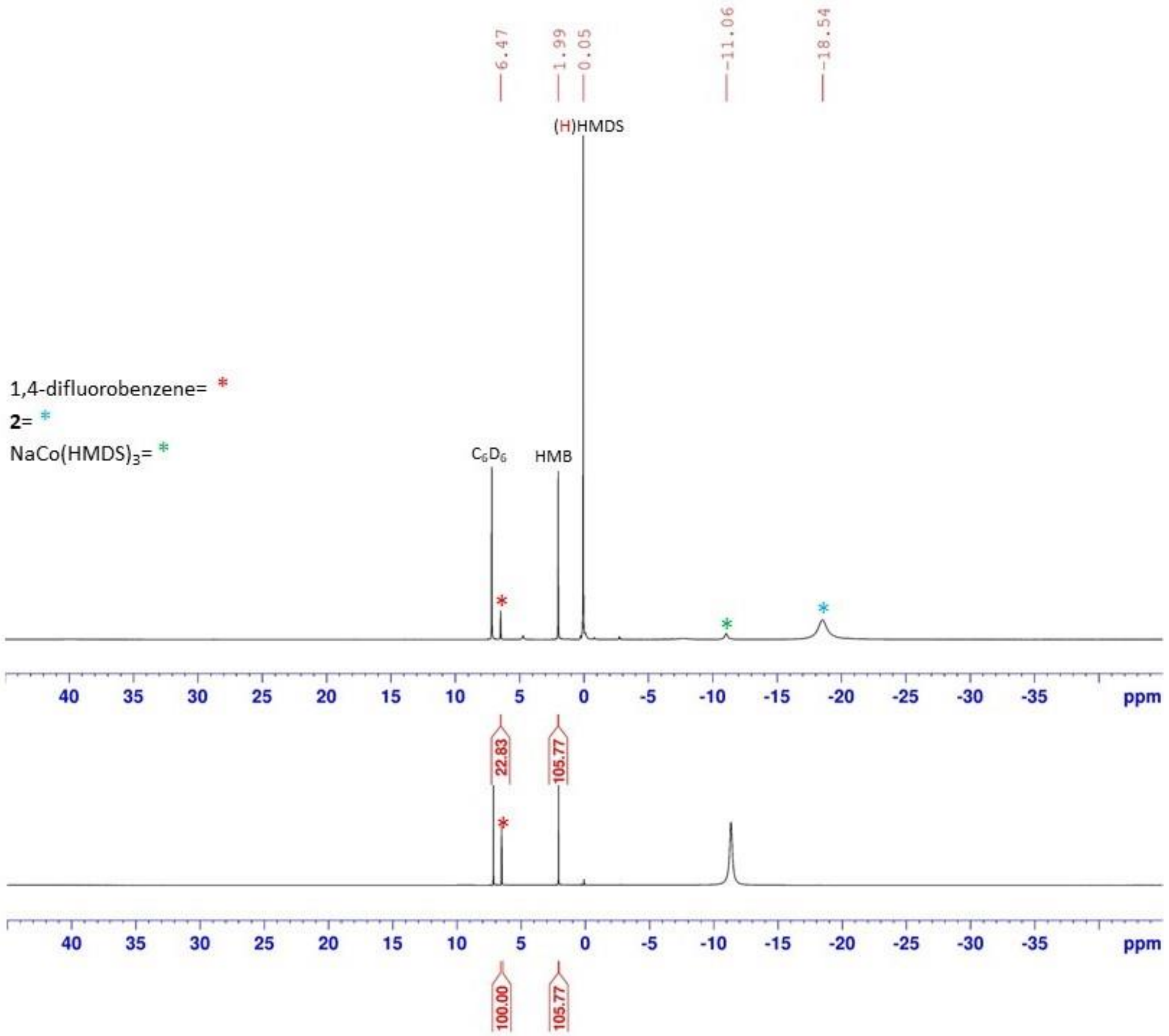

Figure S12. ^1H NMR spectrum in C_6D_6. Conversion of 1,4-difluorobenzene to **2**.

Conversion of 1,3,5-trifluorobenzene to product **3**:

In a J. Young's NMR tube 1,3,5-trifluorobenzene (0.1 mmol, 10 μL) was added together with a constant amount of Hexamethylbenzene as internal standard and dissolved in 0.5 mL of C_6D_6. An initial ^1H NMR was recorded. To the clear solution 2 eq. of $NaCo(HMDS)_3$ (**1**) (0.2 mmol, 0.112 g) was added to obtain a light green solution. A second ^1H NMR was recorded showing a final 67 % conversion of 1,3,5-trifluorobenzene to $[Na_2Co_2(C_6F_3H)(HMDS)_4]$ (**3**).

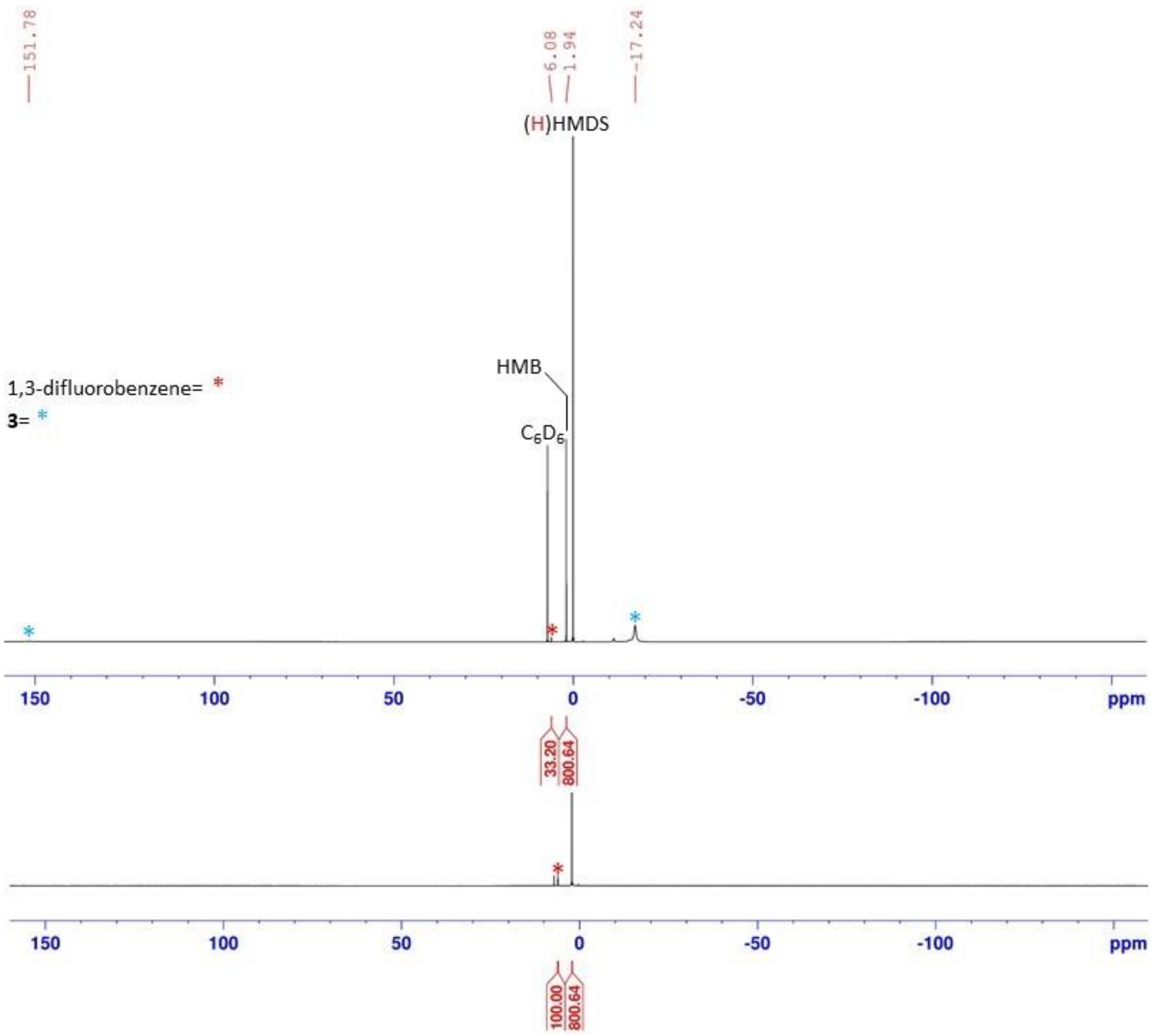

Figure S13. ^1H NMR spectrum in C_6D_6 conversion of 1,3,5-trifluorobenzene to **3**.

Conversion of 1,2,4,5-tetrafluorobenzene to **4**:

In a J. Young's NMR tube 1,2,4,5-tetrafluorobenzene (0.1 mmol, 11 μL) was added together with a constant amount of Hexamethylbenzene as internal standard and dissolved in 0.5 mL of C_6D_6. An initial ^{1}H NMR was recorded. To the clear solution 2 eq. of NaCo(HMDS)$_3$ (**1**) (0.2 mmol, 0.112 g) was added to obtain a light green solution. A second ^{1}H NMR was recorded showing a final 86 % conversion of 1,2,4,5-tetrafluorobenzene to [$Na_2Co_2(C_6F_4)(HMDS)_4$] (**4**).

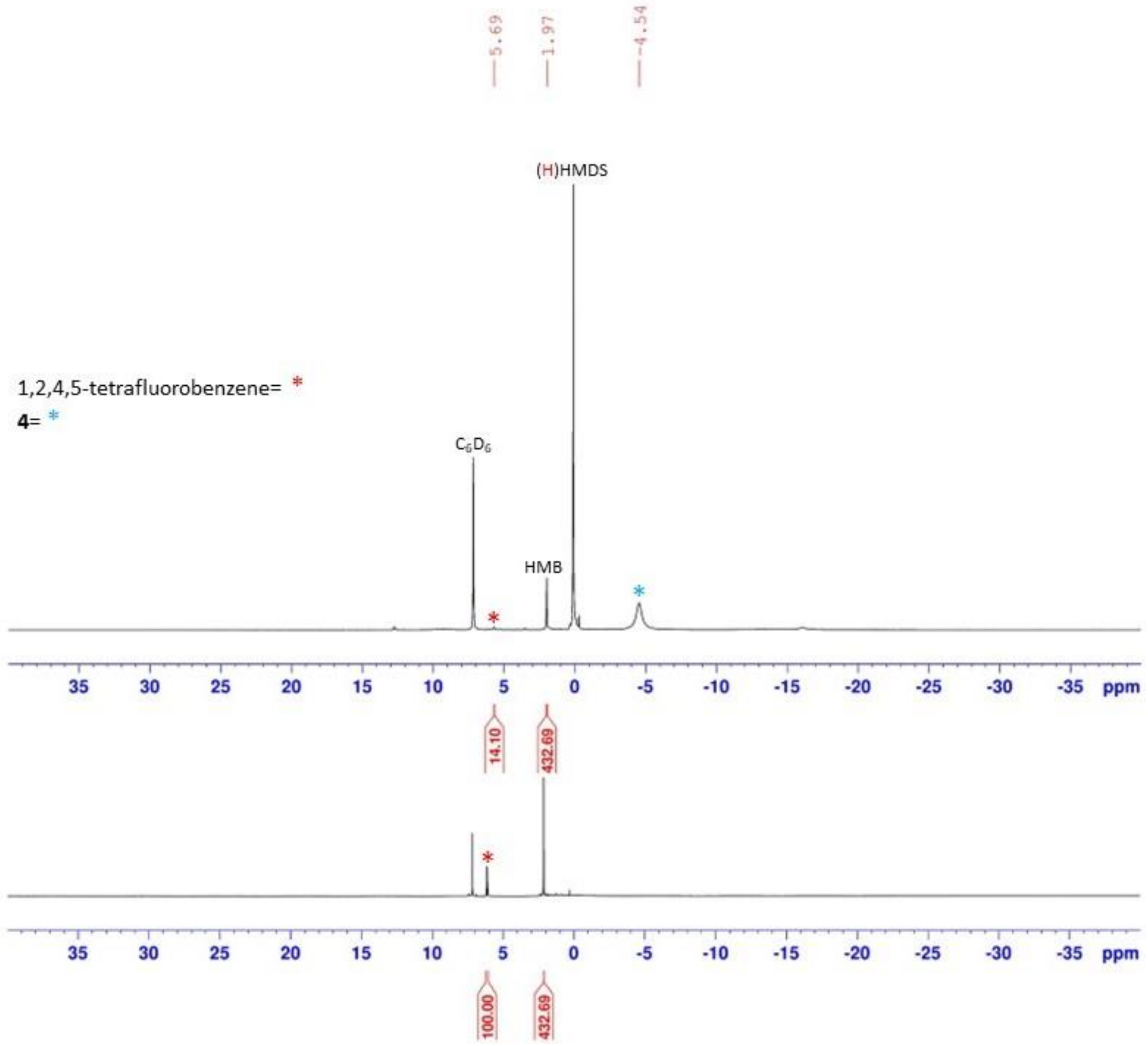

Figure S14. ^{1}H NMR spectrum in C_6D_6 conversion of 1,2,4,5-tetrafluorobenzene to (**4**).

Conversion of 1,2,4,5-tetrafluorobenzene to **5**:

In a J. Young's NMR tube 1,2,4,5-tetrafluorobenzene (0.1 mmol, 11 μL) was added together with a constant amount of Hexamethylbenzene as internal standard and dissolved in 0.5 mL of d_8-Tol. An initial ^{1}H NMR was recorded. The clear solution was cooled to 0 °C and 1 eq. of $NaCo(HMDS)_3$ (**1**) (0.1 mmol, 0.0563 g) was added to obtain a light green solution. A second ^{1}H NMR was recorded showing a final 88 % conversion of 1,2,4,5-tetrafluorobenzene to $[NaCo(HMDS)_2(C_6F_4H)]$ (**5**).

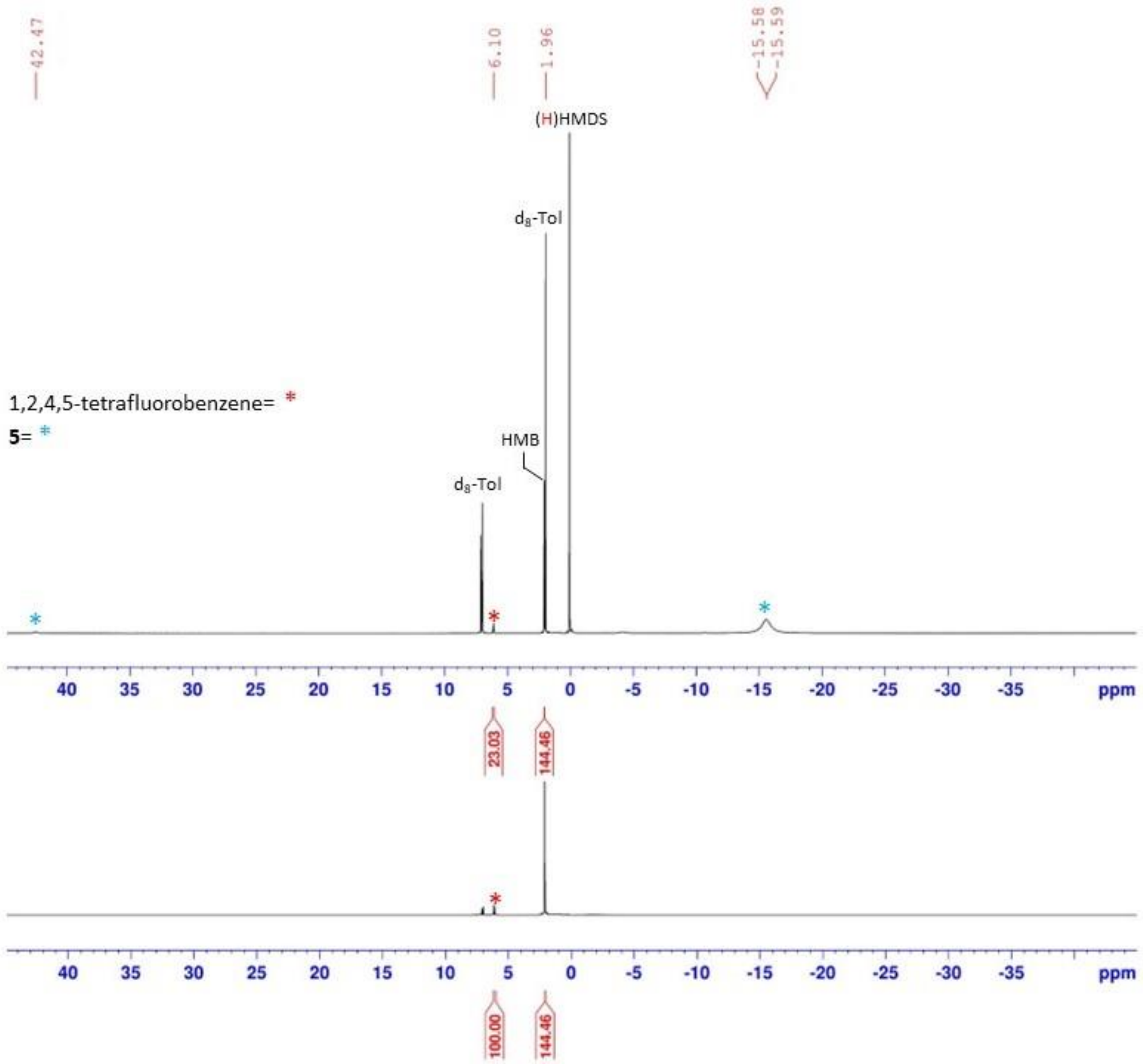

Figure S15. ^{1}H NMR spectrum in d_8-Tol. Conversion of 1,2,4,5-tetrafluorobenzene to (**5**).

Conversion of 1,3,5-trifluorobenzene to **7**:

In a J. Young's NMR tube 1,3,5-trifluorobenzene (0.1 mmol, 10 μL) was added together with a constant amount of Hexamethylbenzene as internal standard and dissolved in 0.5 mL of C_6D_6. An initial ^{1}H NMR was recorded. To the clear solution 3 eq. of $NaCo(HMDS)_3$ (**1**) (0.3 mmol, 0.169 g) was added to obtain a light green solution. After heating for 16 hours at 80 °C a second ^{1}H NMR was recorded showing the full consumption of 1,3,5-trifluorobenzene (signal at 6.08 ppm) to form [1,3-bis(CoHMDS)-2,4,6-tris(HMDS)-C_6H] (**7**) (signals at 10.36, −16.58 and −80.34) and multiple unidentified paramagnetic species.

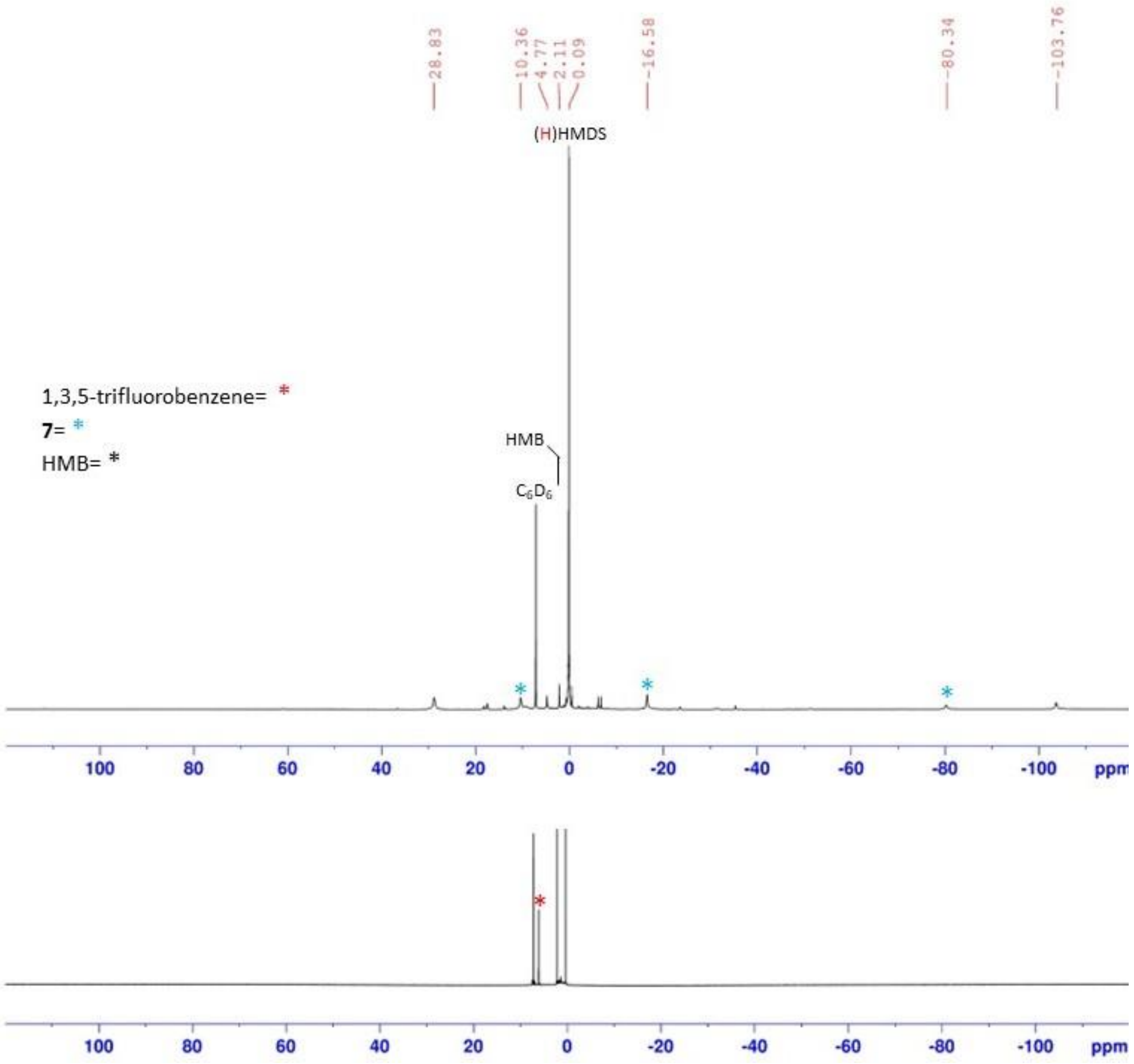

Figure S16. ^{1}H NMR spectrum in C_6D_6. Conversion of 1,3,5-trifluorobenzene to product **7**.

Conversion of 1,3,5-trifluorobenzene to **8**:

In a J. Young's NMR tube 1,3,5-trifluorobenzene (0.1 mmol, 10 μL) was added together with a constant amount of Hexamethylbenzene as internal standard and dissolved in 0.5 mL of C_6D_6. An initial ^{1}H NMR was recorded. To the clear solution 2 eq. of $LiCo(HMDS)_3$ (0.2 mmol, 0.109 g) was added to obtain a light green solution. After heating for 16 hours at 80 °C a second ^{1}H NMR was recorded showing a final 65 % conversion of 1,4-difluorobenzene to $[LiCo(HMDS)_2(C_6F_3H_2)]$ (**8**). The second equivalent of **1** can be observed unreacted in solution (14.27, −12.17 ppm).

NB: $LiCo(HMDS)_3$ was prepared according to a modified literature procedure.[6]

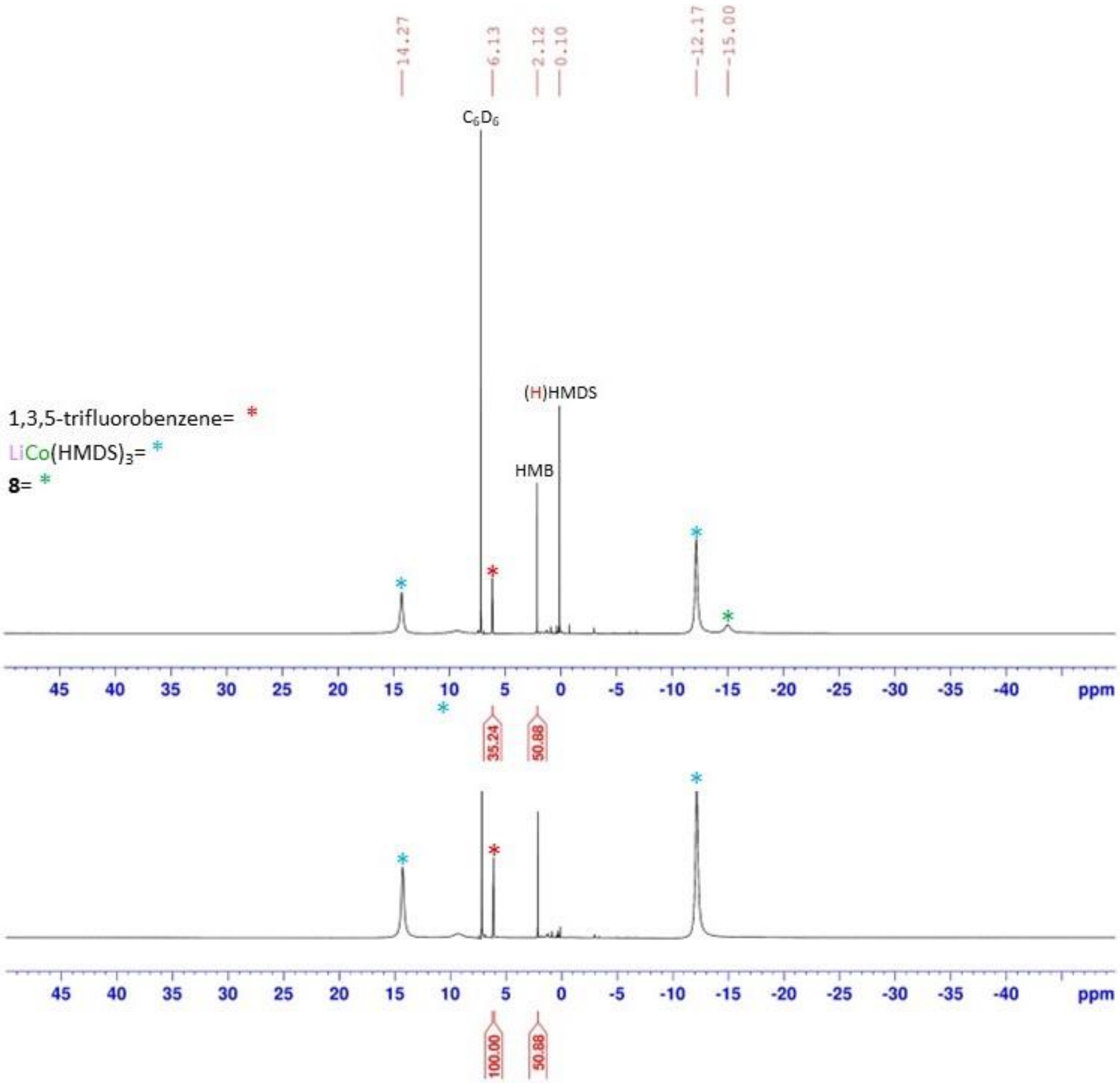

Figure S17. ^{1}H NMR spectrum in C_6D_6. Conversion of 1,3,5-trifluorobenzene to product **8**.

Solution Magnetic Moments Susceptibilities

General method:

In a J. Young's NMR tube, the respective compounds were accurately weighted and the initial mass noted. 0.5 ml of deuterated solvent (C_6D_6 for compounds **2**,**3**,**4**,**7** and **8** and d_8-Tol for compound **5**) was added and the final mass noted. To the clear solution, a sealed capillary containing a mixture of proteo and deuterated solvent (1:50 ratio) was added and ^{1}H NMR spectra recorded on a Bruker Avance III HD 300 spectrometer at 300 K. The effective magnetic moment was calculated considering the peak separation (Δf) of the solvent resonance between that of pure solvent (in the capillary) and that shifted by the paramagnet (outside of the capillary). This can be calculated in ppm and converted into Hz using Eq. 1.1. The magnetic susceptibility and the magnetic moment can then be calculated using Eq. 1.2.

For each compound, the measurement was repeated three times and the effective magnetic moment (μ_{eff}) calculated as average of the calculated values. In brackets, the standard deviation. (see TableS2).

$$\Delta f = \Delta ppm \times F \qquad \text{(Eq. 1.1)}$$

$$\chi_g = \frac{3\Delta f}{4\pi F c} \; ; \; \chi_M = \chi_g \times MW \; ; \; \chi_D \approx -\frac{MW}{2} \times 10^{-6} \; ; \; \chi'_M = \chi_M - \chi_D \; ; \; \boldsymbol{\mu_{eff}} = \sqrt{\mathbf{8}(\boldsymbol{\chi'_M T})} \qquad \text{(Eq. 1.2)}$$

Table S1. Symbols and units in referment to Eq. 1.1 and Eq.1.2.

Symbol	Value	Units
Δf	Frequency shift	Hz
F	Spectrometer frequency	Hz
c	Mass concentration	g cm^{-3}
X_g	Mass susceptibility	cm^3 g^{-1}
MW	Molar mass/molecular weight	g mol^{-1}
X_M	Molar susceptibility	cm^3 mol^{-1}
X_D	Diamagnetic correction	cm^3 mol^{-1}
X_M	Corrected molar susceptibility	cm^3 mol^{-1}
T	Measurement temperature	K
μ_{eff}	Effective magnetic moment	μ_B

Effective magnetic moment calculations:

Table S2. Effective magnetic moment calculations for compounds **2-5**,**7-8**.

Entry	Compound		Solvent	Mass of sample (g)	Mass of sample + solvent (g)	Δf Frequency Shift (Hz)	Mr Molar mass of sample (g mol^{-1})	μ_{eff} Effective magnetic moment (μ_B)	μ_{eff} Average effective magnetic moment (μ_B)
1	[NaCo(HMDS)$_2$(C$_6$F$_2$H$_3$)] (**2**)								4.36 (±0.23)
		n1	C_6D_6	0.0054	0.5691	139.56	587.89	4.22	
		n2	C_6D_6	0.0047	0.5241	161.74	587.89	4.63	
		n3	C_6D_6	0.0068	0.5478	184.66	587.89	4.24	
2	[Na$_2$Co$_2$(C$_6$F$_3$H)(HMDS)$_4$] (**3**)								5.69 (±0.06)
		n1	C_6D_6	0.0109	0.5311	357.54	935.46	5.75	
		n2	C_6D_6	0.0084	0.5137	276.72	935.46	5.69	
		n3	C_6D_6	0.0112	0.5798	322.74	935.46	5.63	
3	[Na$_2$Co$_2$(C$_6$F$_4$)(HMDS)$_4$] (**4**)								5.61 (±0.08)
		n1	C_6D_6	0.0096	0.5411	294.37	953.45	5.67	
		n2	C_6D_6	0.0098	0.4908	331.85	953.45	5.63	
		n3	C_6D_6	0.0109	0.5031	340.48	953.45	5.51	
4	[NaCo(HMDS)$_2$(C$_6$F$_4$H)] (**5**)								4.87 (±0.23)
		n1	d_8-Tol	0.0077	0.5501	285.87	551.76	4.80	
		n2	d_8-Tol	0.0063	0.5157	286.98	551.76	5.14	
		n3	d_8-Tol	0.0074	0.5194	276.88	551.76	4.68	
5	[1,3-bis(CoHMDS)-2,4,6-tris(HMDS)-C$_6$H] (**7**)								5.56 (±0.29)
		n1	C_6D_6	0.0034	0.5254	129.03	801.32	5.71	
		n2	C_6D_6	0.0097	0.5011	168.01	801.32	5.21	
		n3	C_6D_6	0.0032	0.5773	112.10	801.32	5.76	
6	[LiCo(HMDS)$_2$(C$_6$F$_3$H$_2$)] (**8**)								4.30 (±0.27)
		n1	C_6D_6	0.0061	0.5763	172.52	517.72	4.18	
		n2	C_6D_6	0.0041	0.5455	152.79	517.72	4.63	
		n3	C_6D_6	0.0055	0.5703	150.66	517.72	4.11	

Note = The magnetic moments of compounds **3**, **4** and **7** can be explained by the presence of two distorted trigonal high spin Co(II) centres (S = 3/2). The overall slightly higher spin only value from an ideal value of 5.47 µB (calculated considering Spin only value for one Co(II) (S = 3/2) being 3.87 µB and the total (spin only) moment for compounds with two Co centres being 3.87 x √2 = 5.47 µB) can be due to small SOC (spin orbit coupling) contribution. Although, it can not be excluded the presence of weak ferromagnetic coupling between the two metal centres occurring through the aromatic ring.

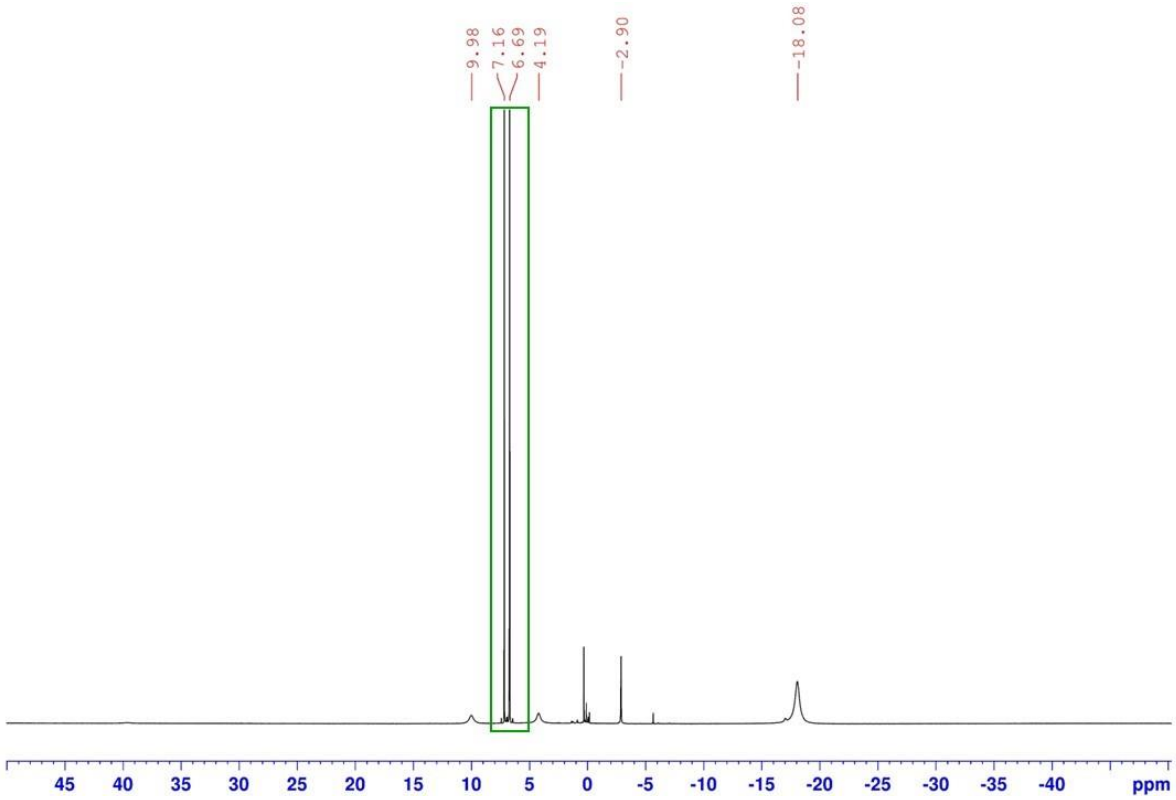

Figure S18. ^1H NMR spectrum of compound **2**, Table1 Entry1_n1. Δ*ppm*= 0.47 ppm, Δ*f*= 139.56 Hz.

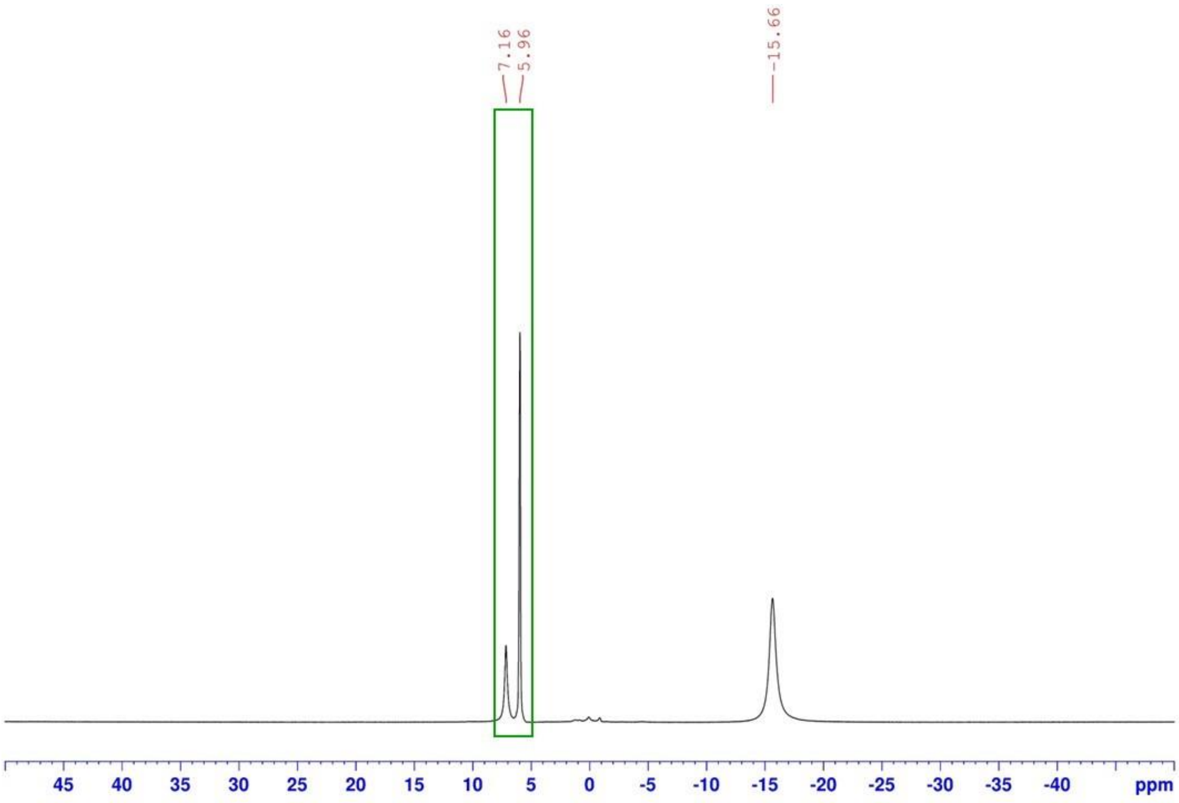

Figure S19. ^1H NMR spectrum of compound **3**, Table1 Entry2_n1. Δ*ppm*= 1.19 ppm, Δ*f*= 357.54 Hz.

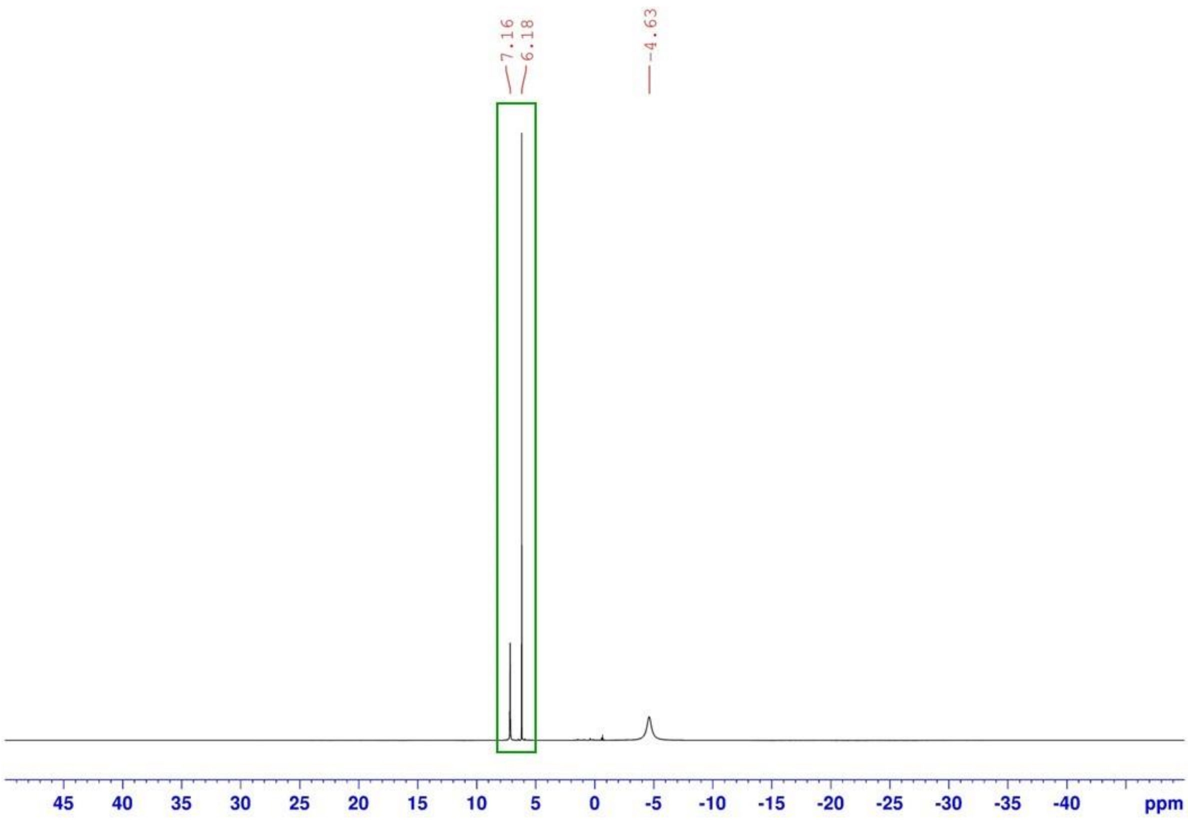

Figure S20. ^{1}H NMR spectrum of compound **4**, Table1 Entry3_n1. Δ*ppm*= 0.98 ppm, Δ*f*= 294.37 Hz.

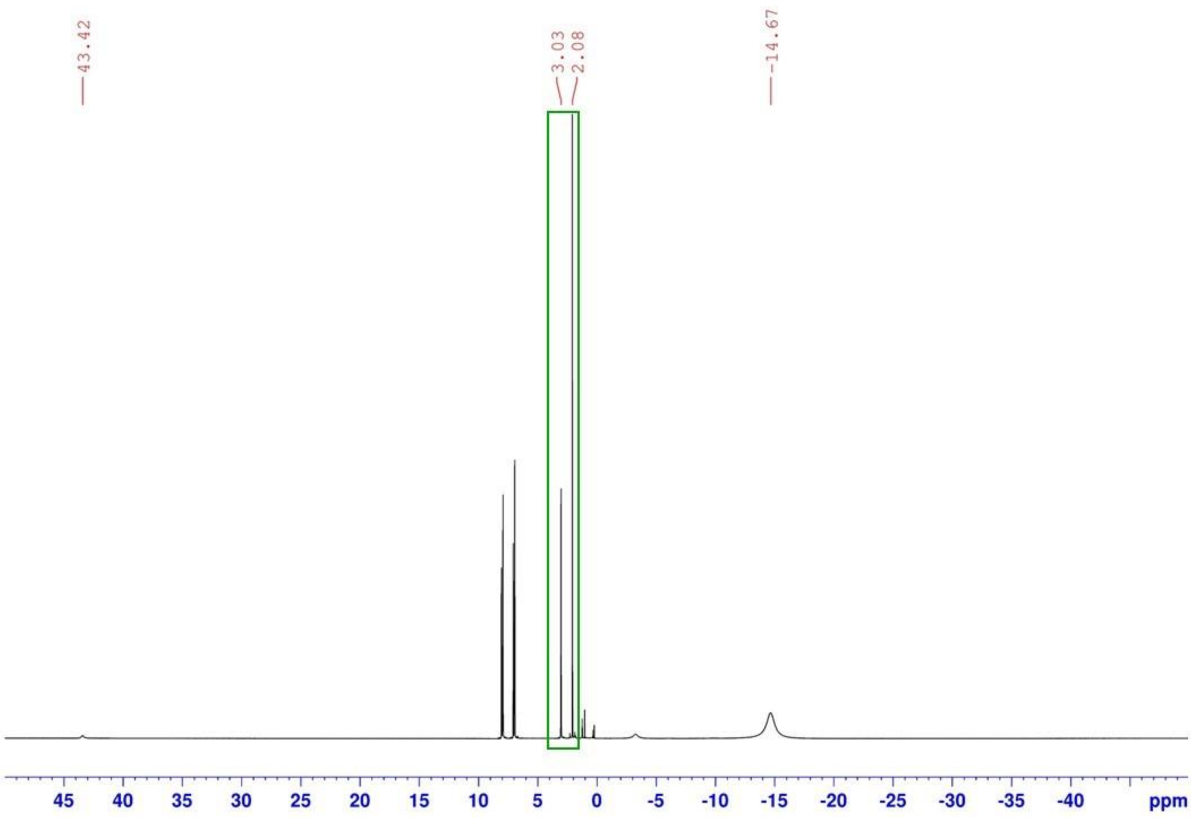

Figure S21. ^{1}H NMR spectrum of compound **5**, Table1 Entry4_n1. Δ*ppm*= 0.95 ppm, Δ*f*= 285.87 Hz.

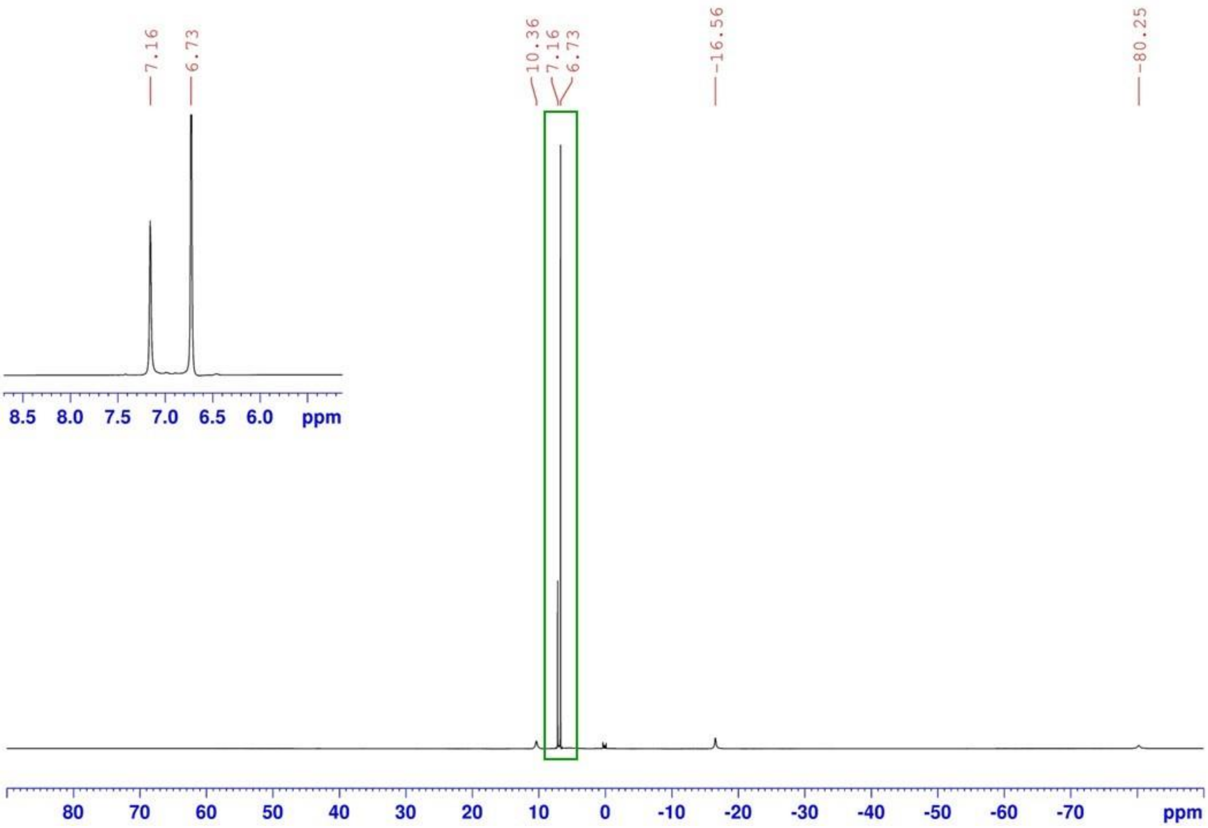

Figure S22. ^{1}H NMR spectrum of compound **7**, Table1 Entry5_n1. Δ*ppm*= 0.43 ppm, Δ*f*= 129.03 Hz.

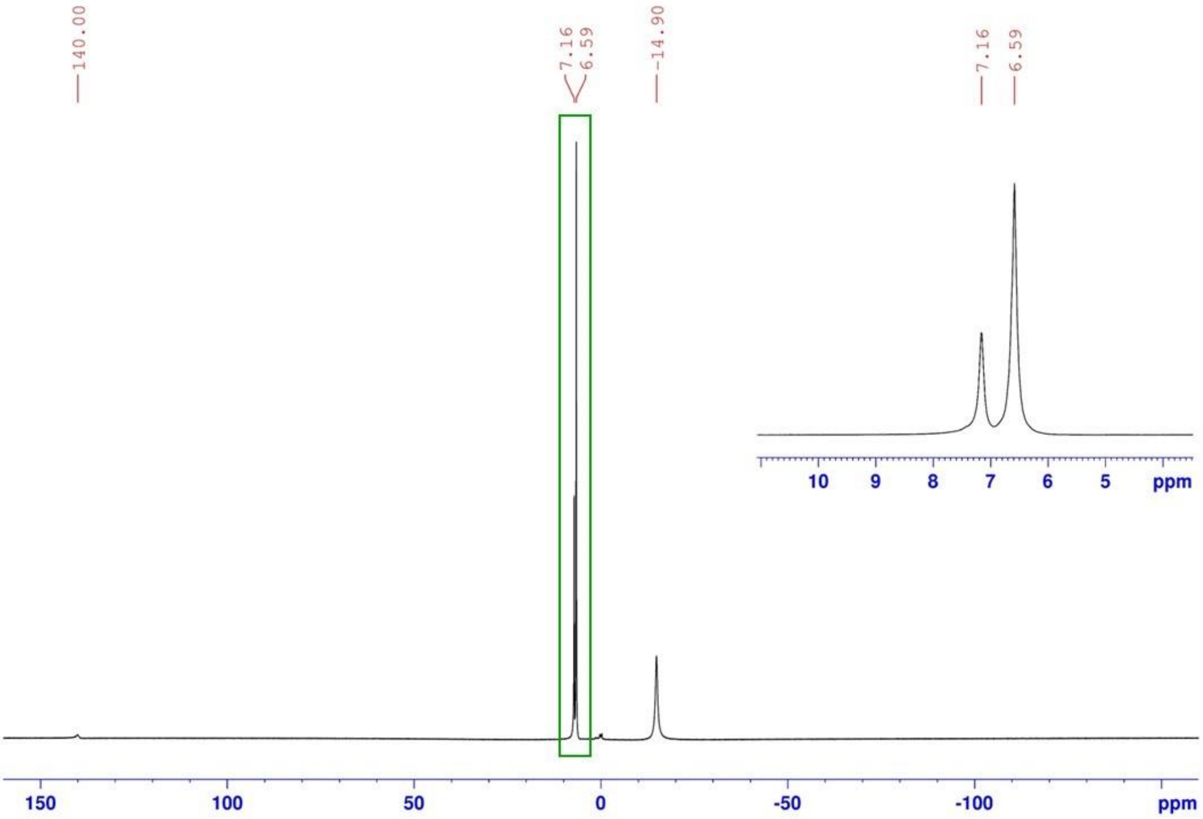

Figure S23. ^{1}H NMR spectrum of compound **8**, Table1 Entry6_n1. Δ*ppm*= 0.57 ppm, Δ*f*= 172.52 Hz.

X-Ray Crystallographic Figures and Data

The crystal structures of compounds **2, 3, 4, 7** and **8** have been deposited into the Cambridge Crystallographic Data Centre (CCDC) and have been assigned the following numbers: 2247797-2247801. Selected crystallographic and refinement parameters are presented in Tables S4, S5 and S6 below. In all cases samples immersed in inert oil were mounted at ambient conditions and transferred into the cold stream of nitrogen.

The structures of compounds **2, 3, 4, 7** and **8** were measured at the University of Bern, Switzerland. All measurements were performed on either a RIGAKU Synergy S or Oxford Diffraction SuperNova area-detector diffractometer using mirror optics monochromated Cu Kα radiation (λ = 1.54184 Å) or Mo Kα radiation (λ = 0.71073 Å), respectively. Data reduction was performed using the CrysAlisPro program.[7] The structure was solved by direct methods using SHELXT,[8] which revealed the positions of all non-hydrogen atoms of the title compound. Refinement of the structure was carried out on F2 using full-matrix least-squares procedures. All calculations were performed using the SHELXL-2014/7 program in Olex2.[8-9]

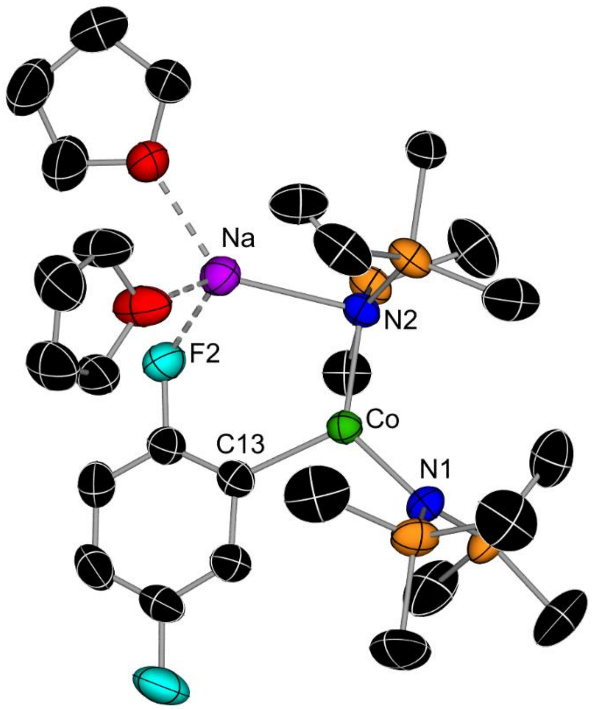

Figure S24. Molecular structure of [NaCo(HMDS)$_2$(C$_6$F$_2$H$_3$)] (**2**). Hydrogen atoms omitted for clarity. Ellipsoids displayed at 50% probability level. Selected bond distances (Å) and angles (°): Co–C13 2.052(6), Co–N1 1.938(1), Co–N2 1.986(7), Na–N2 2.491(3), Na···F2 2.252(6); C13–Co–N1 113.5(2), C13–Co–N2 120.3(4), N1–Co–N2 126.1(2).

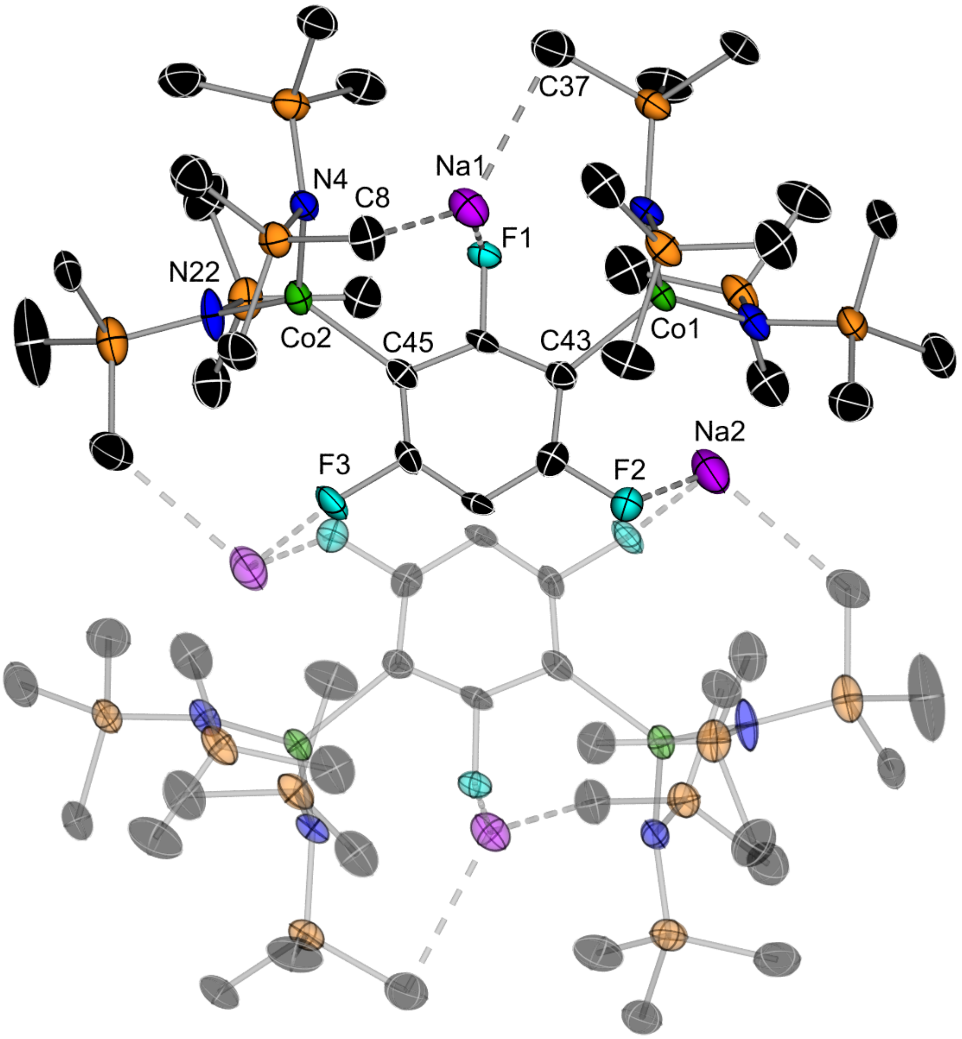

Figure S25. Molecular structure of $[Na_2Co_2(C_6F_3H)(HMDS)_4]$ (**3**). Hydrogen atoms omitted for clarity. Ellipsoids displayed at 50% probability level. Selected bond distances (Å) and angles (°): Co1–C43 2.046(5), Co2–C45 2.053(5), Co2–N4 1.988(4), Co2–N22 1.932(5); Na1···F1 2.244(3), Na1···C8 2.835(5), Na1···C37 2.869(6), Na1···F2 2.302(3); C45–Co2–N22 122.6(6), N22–Co2–N4 127.4(9), N4–Co2–C45 109.8(1).

a)

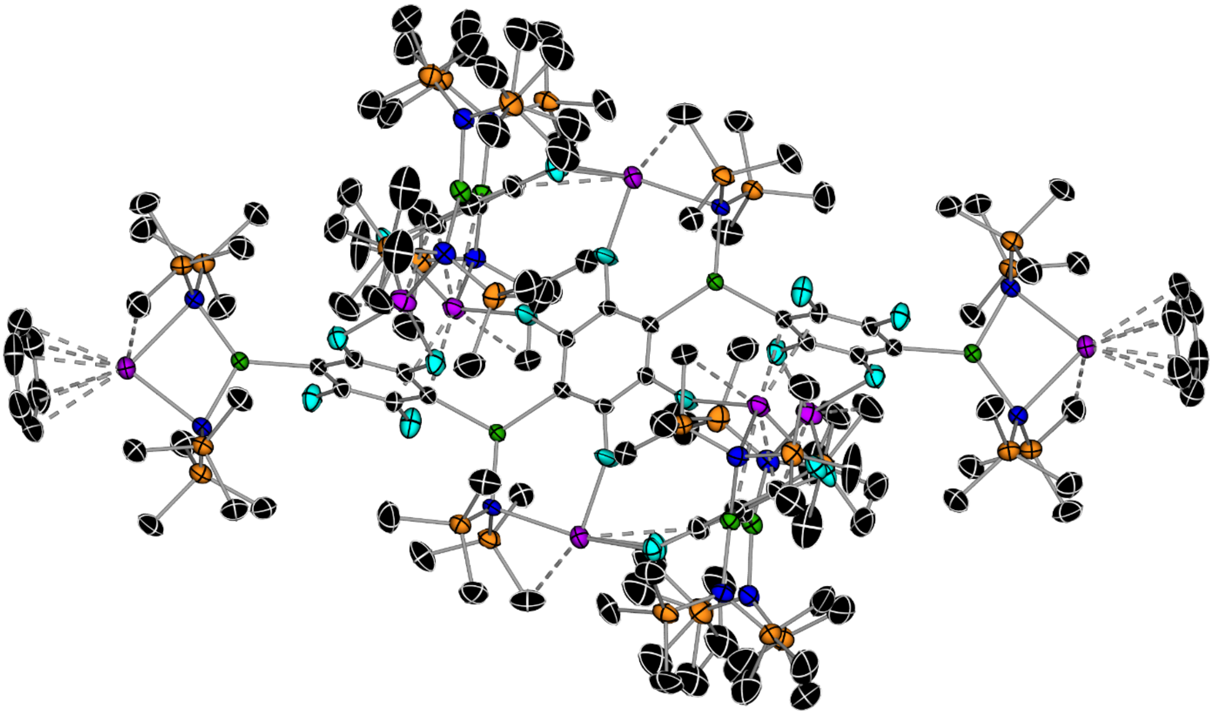

b)

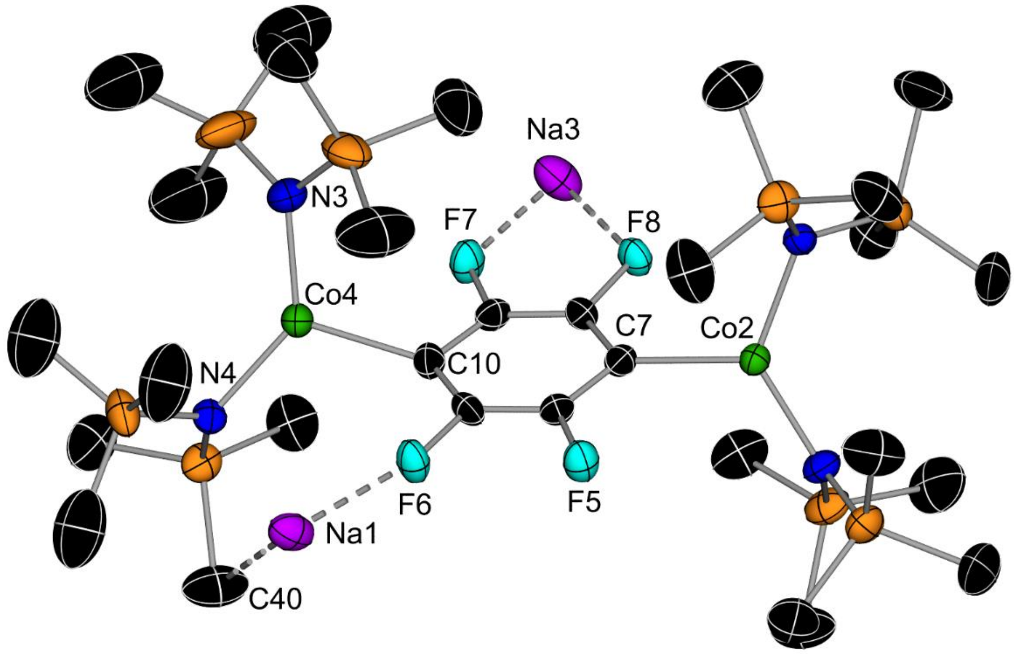

Figure S26. Molecular structure of $[Na_2Co_2(C_6F_4)(HMDS)_4]$ (**4**) as di-solvated pentamer in benzene (a) and its monomeric section (b). Hydrogen atoms omitted for clarity. Ellipsoids displayed at 50% probability level. Selected bond distances (Å) and angles (°): Co2–C7 2.069(5), Co4–C10 2.090(5), Co4–N3 1.921(5), Co4–N4 1.975(4), Na1···F6 2.354(4), Na1···C40 2.959(7), Na3···F7 2.282(4), Na3···F8 2.561(4); C10–Co4–N4 115.4(2), N4–Co4–N3 133.1(8), N3–Co4–C10 110.7(2).

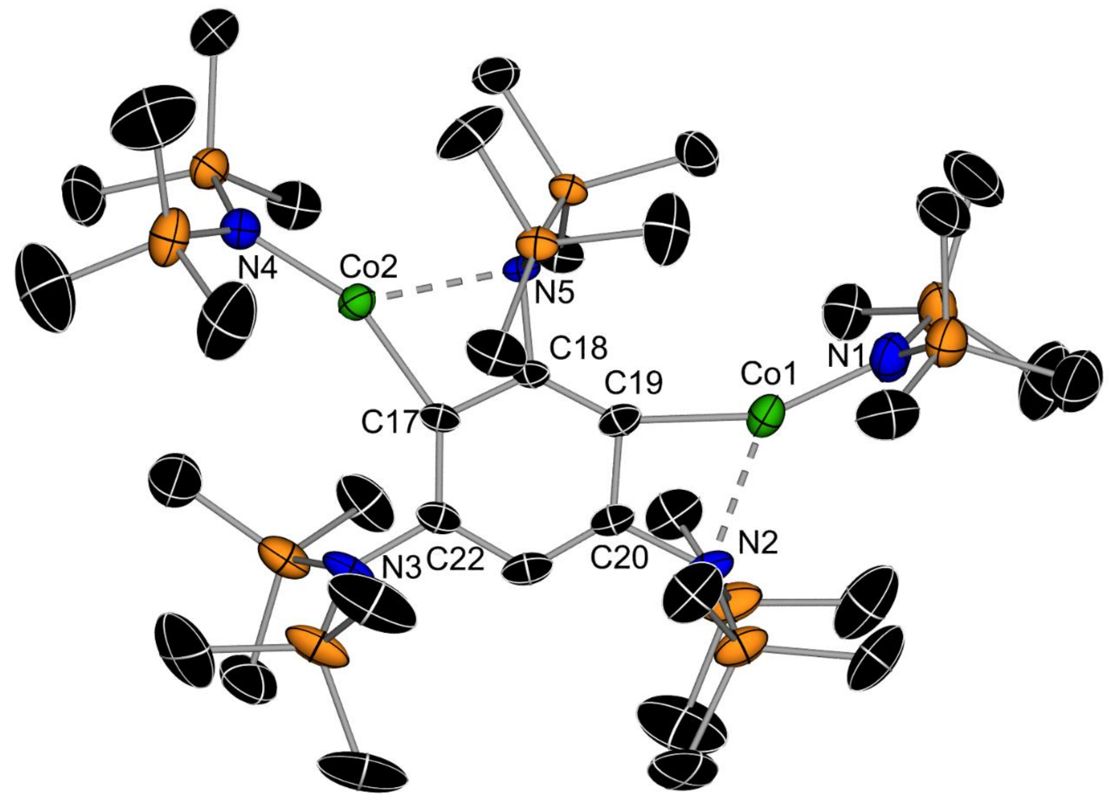

Figure S27. Molecular structure of [1,3-bis(CoHMDS)-2,4,6-tris(HMDS)-C_6H] (**7**). Hydrogen atoms omitted for clarity. Ellipsoids displayed at 50% probability. Selected bond distances (Å) and angles (°): Co1–C19 2.015(7), Co1–N1 1.903(8), Co1–N2 2.281(6), Co2–C17 1.976(7),Co2–N4 1.906(6), Co2–N5 2.340(6), C18–N5 1.495(7), C20–N2 1.501(9), C22–N3 1.441(9); C19–Co1–N1 156.5(3), C17–Co2–N4 156.6(3).

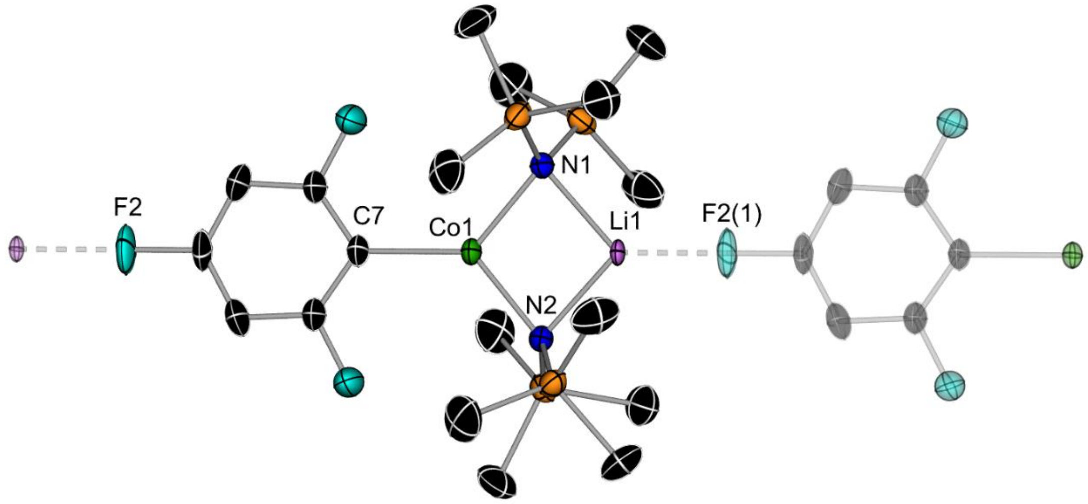

Figure S28. Molecular structure of LiCo(HMDS)$_2$(C$_6$F$_3$H$_2$) (**8**). Hydrogen atoms omitted for clarity. Ellipsoids displayed at 50% probability. Selected bond distances (Å) and angles (°): Co1–C7 2.043(3), Co1–N1 1.998(8), Co1–N2 1.998(8), Li1–N1 2.067(3), Li1–N2 2.067(3); C7–Co1–N1 129.4(4), N1–Co1–N2 101.1(1), N2–Co1–C7 129.4(4).

X-Ray Tables

Table S3. Crystal data and structure refinement parameters for compounds **2, 3, 4, 7** and **8**.

Compound	2	3	4	7	8
CCDC Number	2247797	2247798	2247799	2247800	2247801
Identification code	22EH144_AL579	22EH075_AL491	20EH043_AL46	22EH069_AL453	23EH059_AL639
Empirical formula	$C_{26}H_{55}CoF_2N_2NaO_2Si_4$	$C_{60}H_{146}Co_4F_6N_8Na_4Si_{16}$	$C_{138}H_{276}Co_8F_{20}N_{14}Na_8Si_{28}$	$C_{36}H_{91}Co_2N_5Si_{10}$	$C_{18}H_{38}CoF_3LiN_2Si_4$
Formula weight	660.00	1870.96	3953.59	992.89	517.73
Temperature/K	173.00(10)	173.00(10)	173.01(10)	173.00(10)	173.00(10)
Crystal system	monoclinic	triclinic	monoclinic	monoclinic	orthorhombic
Space group	$P2_1/n$	P-1	$P2_1/c$	$P2_1$	Ccc2
a/Å	17.5676(2)	9.86820(6)	21.6543(2)	9.10611(6)	13.3832(2)
b/Å	11.28947(11)	16.33450(14)	17.95986(12)	29.0863(2)	18.7778(3)
c/Å	20.1652(3)	18.12805(16)	27.5932(3)	32.9649(2)	10.86351(14)
α/°	90	113.0609(8)	90	90	90
β/°	112.3623(14)	102.1123(6)	97.4528(10)	95.7961(6)	90
γ/°	90	98.0739(6)	90	90	90
Volume/Å^3	3698.58(8)	2546.96(4)	10640.59(18)	8686.54(11)	2730.06(7)
Z	4	1	2	6	4
ρ_{calc}g/cm^3	1.185	1.220	1.234	1.139	1.260
μ/mm^{-1}	0.639	7.363	6.925	6.677	0.832
F(000)	1412.0	992.0	4156.0	3216.0	1092.0
Crystal size/mm	0.459 × 0.233 × 0.063	0.213 × 0.19 × 0.142	0.222 × 0.109 × 0.093	0.276 × 0.201 × 0.157	0.162 × 0.11 × 0.076
Radiation/Å	Mo Kα (λ = 0.71073)	Cu Kα (λ = 1.54184)	Cu Kα (λ = 1.54184)	Cu Kα (λ = 1.54184)	Mo Kα (λ = 0.71073)
2Θ range for data collection/°	3.902 to 61.502	5.538 to 137.54	5.886 to 133.202	5.39 to 149.008	4.338 to 67.334
Index ranges	-25 ≤ h ≤ 25, -16 ≤ k ≤ 16, -28 ≤ l ≤ 28	-11 ≤ h ≤ 11, -19 ≤ k ≤ 19, -21 ≤ l ≤ 21	-25 ≤ h ≤ 24, -21 ≤ k ≤ 19, -32 ≤ l ≤ 32	-10 ≤ h ≤ 11, -36 ≤ k ≤ 36, -41 ≤ l ≤ 41	-20 ≤ h ≤ 20, -28 ≤ k ≤ 28, -16 ≤ l ≤ 16
Reflections collected	110235	97018	119030	176467	42315
Independent reflections	11548 [R_{int} = 0.0505, R_{sigma} = 0.0263]	9406 [R_{int} = 0.0385, R_{sigma} = 0.0166]	18807 [R_{int} = 0.0716, R_{sigma} = 0.0478]	35452 [R_{int} = 0.0802, R_{sigma} = 0.0454]	5011 [R_{int} = 0.0312, R_{sigma} = 0.0198]
Parameters	11548/52/512	9406/19/495	18807/156/1041	35452/71/1453	5011/1/141
Goodness-of-fit on F^2	1.032	1.026	1.034	1.078	1.013
Final R indexes [I>=2σ (I)]	R_1 = 0.0402, wR_2 = 0.0973	R_1 = 0.0248, wR_2 = 0.0672	R_1 = 0.0539, wR_2 = 0.1261	R_1 = 0.0722, wR_2 = 0.1989	R_1 = 0.0272, wR_2 = 0.0636
Final R indexes [all data]	R_1 = 0.0617, wR_2 = 0.1078	R_1 = 0.0259, wR_2 = 0.0679	R_1 = 0.0873, wR_2 = 0.1484	R_1 = 0.0767, wR_2 = 0.2051	R_1 = 0.0335, wR_2 = 0.0655

Largest diff. peak/hole / e Å^{-3}	0.29/-0.28	0.33/-0.21	0.78/-0.32	1.66/-0.69	0.36/-0.18
Flack parameter	-	-	-	0.463(4)	0.242(11)

References

1 R. A. Andersen, K. Faegri, J. C. Green, A. Haaland, M. F. Lappert, W. P. Leung, K. Rypdal, *Inorg. Chem.* 1988, **27**, 1782–1786.

2 Konig S.N., Layfield R.A.; *Inorg. Chem.* 2014, **26**, 4302.

3 A. B. Pangborn, M. a. Giardello, R. H. Grubbs, R. K. Rosen, F. J. Timmers, *Organometallics* 1996, **15**, 1518–1520.

4 J. Vela, J. M. Smith, Y. Yu, N. A. Ketterer, C. J. Flaschenriem, R. J. Lachicotte, P. L. Holland, *J. Am. Chem. Soc.* 2005, **127**, 7857–7870.

5 a) D. F. Evans, *J. Chem. Soc.* 1959, **2003**; b) E. M. Schubert, *J. Chem. Educ.* 1992, **69**, 62; c) C. Piguet, *J. Chem. Educ.* 1997, **74**, 815–816.

6 S. Indris, T. Bredow, B. Schwarz and A. Eichhöfer, *Inorg. Chem.*, 2022, **61**, 554–567.

7 CrysAlisPro 1.171.40.37a, Oxford Diffraction Ltd., Yarnton, Oxfordshire, U.K., 2018.

8 G. M. Sheldrick, Acta Crystallogr. Sect. A Found. *Crystallogr.* 2015, **71**, 3–8.

9 O. V. Dolomanov, L. J. Bourhis, R. J. Gildea, J. A. K. Howard, H. Puschmann, *J. Appl. Crystallogr.* 2009, **42**, 339–341.
